# Short-term risk of psychiatric adverse events following COVID-19 vaccination: nationwide self-controlled case series study

**DOI:** 10.1192/bjo.2025.10803

**Published:** 2025-09-22

**Authors:** Hyesung Lee, Bin Hong, Eun Lee, Jin Young Park, Jaehun Jung, Woo Jung Kim, Ju-Young Shin

**Affiliations:** Department of Medical Informatics, Kangwon National University College of Medicine, Chuncheon, Republic of Korea; School of Pharmacy, Sungkyunkwan University, Suwon, Republic of Korea; Department of Psychiatry, Institute of Behavioral Science in Medicine, Yonsei University College of Medicine, Seoul, Republic of Korea; Department of Psychiatry, Severance Hospital, Yonsei University College of Medicine, Seoul, Republic of Korea; Department of Psychiatry, Yongin Severance Hospital, Yonsei University College of Medicine, Yongin, Republic of Korea; Center for Digital Health, Yongin Severance Hospital, Yonsei University Health System, Yongin, Republic of Korea; Department of Preventive Medicine, Korea University College of Medicine, Seoul, Republic of Korea; Institute on Aging, Korea University, Seoul, Republic of Korea; Institute for Innovation in Digital Healthcare, Yonsei University, Seoul, Republic of Korea; Department of Biohealth Regulatory Science, Sungkyunkwan University, Suwon, Republic of Korea; Samsung Advanced Institute for Health Sciences & Technology, Sungkyunkwan University, Seoul, Republic of Korea

**Keywords:** COVID-19 vaccine, psychiatric adverse events, self-controlled case series

## Abstract

**Background:**

To date, little is known about the evidence of a potential risk of psychiatric adverse events following COVID-19 vaccination in large populations with adequate study design.

**Aims:**

To investigate whether COVID-19 vaccination is associated with increased risk of psychiatric adverse events.

**Method:**

We used South Korea’s linkage database to obtain registry data and claims data from 2019 to 2021, and conducted a population-based self-controlled case series study including 11 751 806 individuals. Primary outcomes included anxiety/nervousness, mood disorders, perceptual disturbances/psychoses, aggression/behavioural disturbances, cognitive impairments and sleep disorders within 21 days of COVID-19 vaccination. Secondary outcomes were the stratified primary outcomes according to each individual’s psychiatric history. Conditional Poisson regression was used to estimate incidence rate ratios (IRR) and 95% confidence intervals.

**Results:**

COVID-19 vaccination did not increase the rate of anxiety and nervousness (adjusted IRR 0.95, 95% CI 0.95–0.96), mood disorders (adjusted IRR 0.75, 95% CI 0.75–0.76), perceptual disturbances and psychoses (adjusted IRR 0.72, 95% CI 0.70–0.74), aggression and behavioural disturbances (adjusted IRR 0.93, 95% CI 0.89–0.97), cognitive impairment (adjusted IRR 0.68, 95% CI 0.67–0.69) or sleep disorders (adjusted IRR 0.90, 95% CI 0.89–0.91). Secondary outcomes were consistent with the primary outcome, although the adjusted IRRs for anxiety and nervousness (adjusted IRR 1.17, 95% CI 1.15–1.18) and sleep disorders (adjusted IRR 1.07, 95% CI 1.06–1.09) were statistically significant in individuals with no history of psychiatric disorders. Sensitivity analyses showed consistent results with our main findings.

**Conclusions:**

Our findings provide short-term safety profiles for COVID-19 vaccines regarding psychiatric adverse events. Continuous monitoring of anxiety/nervousness or sleep disorders after COVID-19 vaccination is required regardless of history of psychiatric comorbidities.

The COVID-19 pandemic resulted in a global burden after the outbreak in 2020, and several COVID-19 vaccines were rapidly approved with significant efficacy from randomised controlled trials to respond urgently to the public health crisis.^1–4^ Moreover, subsequent large-scale observational studies have shown real-world effectiveness in the reduction of severe acute respiratory syndrome coronavirus 2 (SARS-CoV-2) infection, transmission, hospital admission and mortality.^5–9^ Based on these findings, more than 13.3 billion doses of COVID-19 vaccines have been administered worldwide,^10^ and vaccination uptake in South Korea was 96.9% in individuals ≥18 years old, who received two or more doses in early 2021.^11^

Along with the solid benefits of the COVID-19 vaccine, there have been reports of serious adverse events, including myocarditis, pericarditis, thrombocytopenic thrombosis, oedema, and Guillain-Barré syndrome, which have raised a broad range of safety concerns.^12,13^ Cardiovascular or neurological adverse events have been widely investigated in diverse population-based studies, but the profile and safety evidence of psychiatric adverse events following COVID-19 vaccination are still limited. Various psychiatric symptoms, including acute confusion, psychosis, mania with psychotic symptoms and depression after COVID-19 vaccination, have been reported in a review study of case reports.^14^ A plausible mechanism is an immune response similar to SARS-CoV-2 infection, causing psychiatric adverse events;^15^ previous studies reported that SARS-CoV-2 infection was related to analogous symptoms, including confusion, anxiety, mood change, insomnia and hallucinations.^16^ There is a meta-analysis suggesting no significant associations of depression and anxiety with COVID-19 vaccination (depression: odds ratio 0.88, 95% CI 0.75–1.03; anxiety: odds ratio 0.86, 95% CI 0.71–1.05).^17^ However, the largest study population (94.7%) in this meta-analysis was a cross-sectional design, and the definition of follow-up periods to identify study outcomes varied between studies from 2 weeks to 6 months. These limitations suggest a possible reverse association or detection bias induced by inconsistent measurement definitions of the study outcomes. Moreover, it appears that scale-oriented studies are not suitable for capturing the full spectrum of various anxiety symptoms, or that they could potentially underestimate clinical anxiety symptoms following vaccination.

Accordingly, we conducted a population-based, self-controlled case series study using a national vaccination registry database linked with claims data, to investigate whether COVID-19 vaccination was associated with an increased risk of developing anxiety and nervousness, mood disorders, perceptual disturbances and psychoses, aggression and behavioural disturbances, cognitive impairments and sleep disorders.

## Method

### Data source

We used a nationwide linkage database, the Korea Disease Control and Prevention Agency-COVID-19-National Health Insurance Service cohort (K-COV-N cohort), which contained the registry data for COVID-19 infection and vaccination from the Korea Disease Control and Prevention Agency (KCDA) and claims data from the National Health Insurance Service (NHIS) from July 2019 to December 2021. The registry data from the KCDA includes all information regarding SARS-CoV-2 infection (e.g. date of confirmation, institution of reporting, pathway of infection and cause of infection) and vaccination (e.g. date of vaccination, type of vaccination and order of vaccination) from 21 February 2021 to 31 December 2021. From the registry data, we identified four types of vaccines: BNT162b2, mRNA-1273, ChAdOx1 nCoV-19 and Ad26.COV2-S.

Claims data in South Korea is a reliable source for records of diagnostic codes (described using the ICD-10) and prescriptions (described using the Anatomical Therapeutic Chemical (ATC) code) in out-patient, in-patient and ambulatory settings. This database represents the entire population of South Korea, and investigators can identify every individual healthcare service provided based on the fee-for-service system.^18^ In this study, over 11 million individuals were included through age and gender random sampling, representing 24% of the entire population. A maximum limit on the volume of data that can be provided to researchers is determined by the data provision policy in South Korea; therefore, 24% of the sampled data was available during the study period.

### Study design and population

We conducted a self-controlled case series study to investigate whether COVID-19 vaccination increased the risk of psychiatric disorders. This study design was originally developed for vaccine safety surveillance, and the key feature is to use each individual as their own control, to automatically minimise time-invariant confounders.^19^ Therefore, the relative risks are estimated by comparing the incidence rate of the outcome of interest between the risk and baseline periods, with adjustment of time-variant confounders if needed.

We first included all individuals identified from our linkage database from 21 February 2021 to 31 December 2021. Individuals were excluded based on the following exclusion criteria: (a) individuals with history of COVID-19 infection before the start date of the study period and (b) individuals who were vaccinated with the first dose after the end date of the study period or who could not be followed up for at least 30 days after the first vaccination. Finally, we included all individuals diagnosed with psychiatric disorders, including anxiety and nervousness, mood disorders, perceptual disturbances and psychoses, aggression and behavioural disturbances, cognitive impairments and sleep disorders. Consequently, we included all individuals with a history of vaccination and outcomes of interest, and six cohorts were generated separately, according to each psychiatric disorder.

### Vaccination

Our exposures of interest were all doses of COVID-19 vaccines, including BNT162b2, mRNA-1273, ChAdOx1 nCoV-19 and Ad26.COV2-S. All individuals were followed up from 21 February 2021 to 31 December 2021, or the date of death, whichever came first. The study period was divided into three separate periods (Supplementary Fig. 1 available at https://doi.org/10.1192/bjo.2025.10803): (a) the 21 day pre-vaccination period, (b) the 21 day post-vaccination period and (c) the baseline period. We defined the pre-vaccination period to evaluate whether the risk of psychiatric disorders increased before vaccination, which could induce potential bias caused by reverse causality when estimating the respective risk in the post-vaccination period.

### Outcomes

We defined six psychiatric disorders as outcomes of interest: (a) anxiety and nervousness, (b) mood disorders, (c) perceptual disturbances and psychoses, (d) aggression and behavioural disturbances, (e) cognitive impairments and (f) sleep disorders. We included only the first occurrence of outcomes in this study, because subsequent outcomes were not independent of the preceding outcomes. We identified these outcomes, using the records of diagnostic codes (Supplementary Table 1).

### Statistical analyses

We described the baseline characteristics of the study population, using means (s.d.) for continuous variables and numbers (percentages) for categorical variables. Gender, age, income level and residential location were assessed at the start of the study period, and vaccination status was assessed throughout the study period. Psychiatric history was assessed using all records of diagnostic codes preceding 1 year from the start date of the study period.

We calculated the incidence rates (per 1000 person-days) and incidence rate ratios with corresponding 95% CIs, using a conditional Poisson regression model to evaluate whether COVID-19 vaccination increased the risk of psychiatric disorders. Incidence rate ratios were calculated using the baseline period as reference; seasonal effects in quarterly categories and the status of COVID-19 infection during the study period were adjusted to account for time-variant confounders. Primary estimates included the incidence rate ratios for individual outcomes and secondary estimates included the stratified incidence rate ratios for individual outcomes according to the history of psychiatric disorder. We further conducted several stratified analyses to investigate the differences in the risk of psychiatric disorders following COVID-19 vaccination across subpopulations based on gender, age group (≤19, 20–45, 46–65 and >65 years), income level, residential location, type of platform (messenger RNA (mRNA) and viral vector vaccination), type of vaccine (BNT162b2, mRNA-1273, ChAdOx1 nCoV-19 and Ad26.COV2-S) and vaccination doses (first, second, third).

We performed three sensitivity analyses to assess the robustness of our findings. First, we excluded individuals who died during the study period, to test one of the assumptions of a self-controlled case series design, in which an event should not affect follow-up during the study period. Second, as COVID-19 is a known risk factor for psychiatric disorders, we excluded individuals who were diagnosed with COVID-19 during the study period, which was adjusted with the time-varying approach in the main analyses. Third, we redefined the post-vaccination period into three separate periods of 7 days (<7, 7–14 and >15 days), to investigate whether the risks of psychiatric disorders following COVID-19 vaccination are different during the risk period.

All statistical analyses were performed with SAS Enterprise Guide 7.1 for Windows (SAS Institute, Cary, North Carolina, USA; https://www.sas.com/). A two-tailed *P* < 0.05 was considered significant. This study was approved by the Institutional Review Board of Sungkyunkwan University, Suwon, South Korea (approval number 2021-04-005). The requirement for informed consent was waived because the study used anonymous administrative data.

## Results

### Baseline characteristics

**Fig. 1:**
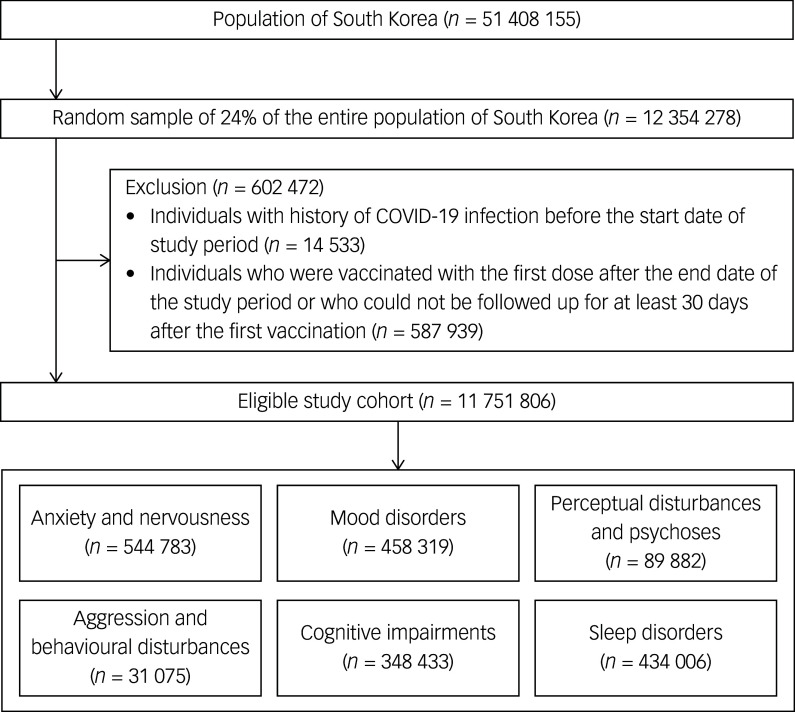
Selection of the study cohort.

Our study cohort included 11 751 806 individuals during the study period (Fig. 1). Among our interest of outcomes, most of the cases were anxiety and nervousness (544 783), followed by mood disorders (458 319), sleep disorders (434 006), cognitive impairments (348 433), perceptual disturbances and psychoses (89 882), and aggression and behavioural disturbances (31 075) (Table 1). All psychiatric disorders showed higher proportion in women (54.3–67.9%), except for aggression and behavioural disturbances (43.7%). The cohorts of aggression and behavioural disturbances included the youngest cases (mean age 30.6 years, s.d. 18.0), and those of cognitive impairment included the oldest cases (mean age 76.9 years, s.d. 11.1). The range of mean age in cases with other outcomes was from 53.0 to 58.5 years. The coverage of COVID-19 vaccination in cases with each study outcome was 87.9–94.2%, and the proportion of the history of psychiatric disorder was the highest in cases with perceptual disturbances and psychoses (84.7%), followed by cognitive impairments (79.7%), mood disorders (76.9%), and aggression and behavioural disturbances (73.7%).

**Table 1:** Baseline characteristics of the study cohort

Characteristic	Anxiety and nervousness	Mood disorders	Perceptual disturbances and psychoses
	(*n* = 544 783)	(*n* = 458 319)	(*n* = 89 882)
Female, *n* (%)	342 317 (62.8)	291 842 (63.7)	48 829 (54.3)
Age, *n* (%)	mean 54.2 (s.d. 19.0)	mean 53.4 (s.d. 20.5)	mean 53.0 (s.d. 17.7)
≤19 years	16 013 (2.9)	16 826 (3.7)	1946 (2.2)
20–45 years	162 836 (29.9)	151 894 (33.1)	27 709 (30.8)
46–65 years	195 960 (36.0)	144 229 (31.5)	39 162 (43.6)
>65 years	169 974 (31.2)	145 370 (31.7)	21 065 (23.4)
Income level, *n* (%)			
First quartile	147 358 (27.0)	142 732 (31.1)	44 007 (49.0)
Second quartile	103 249 (19.0)	83 429 (18.2)	12 879 (14.3)
Third quartile	115 149 (21.1)	89 714 (19.6)	13 311 (14.8)
Fourth quartile	179 027 (32.9)	142 444 (31.1)	19 685 (21.9)
Rural, *n* (%)	239 315 (43.9)	253 251 (55.3)	50 100 (55.7)
Vaccinated, *n* (%)	509 136 (93.5)	424 927 (92.7)	80 589 (89.7)
History of any psychiatric conditions	358 447 (65.8)	352 410 (76.9)	76 101 (84.7)

	Aggression and behavioural disturbances	Cognitive impairments	Sleep disorders
	(*n* = 31 075)	(*n* = 348 433)	(*n* = 434 006)
Female, *n* (%)	13 575 (43.7)	236 637 (67.9)	257 223 (59.3)
Age, *n* (%)	mean 30.6 (s.d. 18.0)	mean 76.9 (s.d. 11.1)	mean 58.5 (s.d. 17.1)
≤19 years	10 060 (32.4)	380 (0.1)	3304 (0.8)
20–45 years	15 601 (50.2)	3960 (1.1)	95 778 (22.1)
46–65 years	3004 (9.8)	46 758 (13.4)	174 504 (40.2)
>65 years	2370 (7.6)	297 335 (85.3)	160 420 (37.0)
Income level, *n* (%)			
First quartile	8602 (27.7)	110 072 (31.6)	124 179 (28.6)
Second quartile	5797 (18.7)	48 981 (14.1)	79 754 (18.4)
Third quartile	5975 (19.2)	61 284 (17.6)	87 750 (20.2)
Fourth quartile	10 701 (34.4)	128 096 (36.8)	142 323 (32.8)
Rural, *n* (%)	14 992 (48.2)	212 968 (61.1)	233 123 (53.7)
Vaccinated, *n* (%)	27 295 (87.9)	314 062 (90.1)	408 738 (94.2)
History of any psychiatric conditions	22 901 (73.7)	277 582 (79.7)	263 875 (60.8)

### Risk of psychiatric disorders following COVID-19 vaccination

The range of incidence rate within 21 days after COVID-19 vaccination for all study outcomes was from 1.54 (perceptual disturbances and psychoses) to 2.41 (anxiety and nervousness) per 1000 person-days (Table 2), which was lower than incidence rate of the baseline period. We observed no association between COVID-19 vaccination and six psychiatric disorders (adjusted incidence rate ratios: anxiety and nervousness 0.95, 95% CI 0.95–0.96; mood disorders 0.75, 95% CI 0.75–0.76; perceptual disturbances and psychoses 0.72, 95% CI 0.70–0.74; aggression and behavioural disturbances 0.93, 95% CI 0.89–0.97; cognitive impairments 0.68, 95% CI 0.67–0.69; sleep disorders 0.90, 95% CI 0.89–0.91).

**Table 2:** Risk of psychiatric adverse events following COVID-19 vaccination in South Korea

Outcome	Number of events	Incidence rate^a^ (95% CI)	Incidence rate ratio^b^ (95% CI)
Anxiety and nervousness			
Baseline	429 906	3.58 (3.57–3.59)	1.00 (Reference)
Pre-vaccination	54 449	2.35 (2.33–2.37)	0.82 (0.82–0.83)
Post-vaccination	60 428	2.41 (2.39–2.43)	0.95 (0.95–0.96)
Mood disorders			
Baseline	381 345	3.75 (3.74–3.76)	1.00 (Reference)
Pre-vaccination	39 767	2.09 (2.07–2.11)	0.76 (0.75–0.76)
Post-vaccination	37 207	1.78 (1.76–1.80)	0.75 (0.75–0.76)
Perceptual disturbances and psychoses			
Baseline	72 161	3.59 (3.56–3.61)	1.00 (Reference)
Pre-vaccination	11 564	3.15 (3.10–3.21)	1.25 (1.23–1.28)
Post-vaccination	6157	1.54 (1.50–1.58)	0.72 (0.70–0.74)
Aggression and behavioural disturbances			
Baseline	26 703	3.61 (3.56–3.65)	1.00 (Reference)
Pre-vaccination	2026	2.04 (1.96–2.14)	0.90 (0.86–0.94)
Post-vaccination	2346	1.94 (1.86–2.02)	0.93 (0.89–0.97)
Cognitive impairments			
Baseline	267 006	3.56 (3.55–3.57)	1.00 (Reference)
Pre-vaccination	42 935	2.84 (2.81–2.87)	0.74 (0.74–0.75)
Post-vaccination	38 492	2.19 (2.17–2.21)	0.68 (0.67–0.69)
Sleep disorders			
Baseline	343 440	3.66 (3.64–3.67)	1.00 (Reference)
Pre-vaccination	43 385	2.23 (2.21–2.26)	0.76 (0.76–0.77)
Post-vaccination	47 181	2.27 (2.25–2.29)	0.90 (0.89–0.91)

Pre-vaccination and post-vaccination were defined as 21 days before and after the vaccination date, respectively.

a Incidence rate was calculated per 1000 person-days.

b Season and infection of COVID-19 were adjusted with a time-varying approach.

We observed higher ranges of incidence rate for our study outcomes in cases without psychiatric history compared with those with psychiatric history (without psychiatric history: 2.99–3.65; with psychiatric history: 1.25–1.93) (Table 3). However, when compared with the baseline period, the risks of mood disorders, perceptual disturbances and psychoses, and aggression and behavioural disturbances 21 days after COVID-19 vaccination were not different, regardless of previous psychiatric conditions. In contrast, when stratifying according to psychiatric history, the risks of anxiety and nervousness, cognitive impairments and sleep disorders differed (adjusted incidence rate ratios with versus without psychiatric history: anxiety and nervousness, 0.79 (95% CI 0.78–0.79) *v*. 1.17 (95% CI 1.15–1.18); cognitive impairments, 0.79 (95% CI 0.78–0.79) *v*. 1.17 (95% CI 1.15–1.18); sleep disorder, 0.79 (95% CI 0.78–0.79) *v*. 1.17 (95% CI 1.15–1.18)).

**Table 3:** Risk of psychiatric adverse events following COVID-19 vaccination in South Korea, stratified by psychiatric history

Outcome	With psychiatric history			Without psychiatric history		
	Number of events	Incidence rate^a^ (95% CI)	Incidence rate ratio^b^ (95% CI)	Number of events	Incidence rate^a^ (95% CI)	Incidence rate ratio^b^ (95% CI)
Anxiety and nervousness						
Baseline	298 273	3.80 (3.78–3.81)	1.00 (Reference)	131 633	3.17 (3.15–3.19)	1.00 (Reference)
Pre-vaccination	30 120	1.95 (1.93–1.97)	0.69 (0.69–0.70)	24 329	3.15 (3.11–3.19)	1.00 (0.99–1.02)
Post-vaccination	30 054	1.79 (1.77–1.81)	0.79 (0.78–0.79)	30 374	3.65 (3.61–3.69)	1.17 (1.15–1.18)
Mood disorders						
Baseline	303 384	3.90 (3.89–3.92)	1.00 (Reference)	77 961	3.25 (3.23–3.27)	1.00 (Reference)
Pre-vaccination	26 528	1.78 (1.76–1.81)	0.66 (0.65–0.66)	13 239	3.20 (3.15–3.26)	0.98 (0.96–1.00)
Post-vaccination	22 498	1.38 (1.36–1.40)	0.64 (0.63–0.65)	14 709	3.20 (3.15–3.26)	0.98 (0.96–1.00)
Perceptual disturbances and psychoses						
Baseline	62 072	3.65 (3.62–3.68)	1.00 (Reference)	10 089	3.25 (3.19–3.32)	1.00 (Reference)
Pre-vaccination	9802	3.15 (3.09–3.21)	1.27 (1.25–1.30)	1762	3.18 (3.04–3.33)	1.00 (0.95–1.05)
Post-vaccination	4227	1.25 (1.21–1.28)	0.60 (0.59–0.62)	1930	3.21 (3.07–3.35)	1.03 (0.98–1.08)
Aggression and behavioural disturbances						
Baseline	20 549	3.76 (3.71–3.81)	1.00 (Reference)	6154	3.18 (3.10–3.26)	1.00 (Reference)
Pre-vaccination	1136	1.57 (1.48–1.67)	0.85 (0.80–0.90)	890	3.31 (3.10–3.53)	0.99 (0.93–1.07)
Post-vaccination	1216	1.37 (1.29–1.45)	0.84 (0.80–0.90)	1130	3.51 (3.31–3.72)	1.05 (0.99–1.12)
Cognitive impairments						
Baseline	217 490	3.64 (3.62–3.65)	1.00 (Reference)	49 516	3.26 (3.23–3.29)	1.00 (Reference)
Pre-vaccination	33 093	2.76 (2.73–2.79)	0.67 (0.67–0.68)	9842	3.14 (3.07–3.20)	0.97 (0.95–0.99)
Post-vaccination	26 999	1.93 (1.91–1.95)	0.57 (0.56–0.58)	11 493	3.22 (3.16–3.28)	1.02 (1.00–1.04)
Sleep disorders						
Baseline	217 288	3.81 (3.80–3.83)	1.00 (Reference)	126 152	3.41 (3.39–3.43)	1.00 (Reference)
Pre-vaccination	23 315	1.97 (1.95–2.00)	0.67 (0.66–0.67)	20 070	2.64 (2.60–2.68)	0.89 (0.87–0.90)
Post-vaccination	23 272	1.83 (1.80–1.85)	0.76 (0.75–0.77)	23 909	2.99 (2.95–3.03)	1.07 (1.06–1.09)

Pre-vaccination and post-vaccination were defined as 21 days before and after the vaccination date, respectively.

a Incidence rate was calculated per 1000 person-days.

b Season and infection of COVID-19 were adjusted with a time-varying approach.

### Subgroup and sensitivity analyses

Most results from the subgroup analyses by gender, age, income level, residential location and type of platform were largely consistent with the main findings (Table 4, Supplementary Tables 2–7). However, the adjusted incidence rate ratios of anxiety and nervousness and sleep disorders were higher in individuals vaccinated with viral vector vaccines only than those vaccinated with mRNA vaccines only (viral vector only versus mRNA only: anxiety and nervousness, 1.21 (95% CI 1.16–1.25) *v*. 0.93 (95% CI 0.92–0.94); sleep disorders, 1.18 (95% CI 1.13–1.23) *v.* 0.86 (95% CI 0.85–0.87)). In addition, the risks of anxiety and nervousness, cognitive impairments and sleep disorders were slightly increased in the middle age group (46–65 years old). The stratified results by individual dose were largely consistent with our main findings, indicating that there were no dose-specific effects on psychiatric outcomes (Supplementary Table 8).

**Table 4:** Stratified analyses for the risk of psychiatric adverse events following COVID-19 vaccination in South Korea

Subgroup	Incidence rate ratio^a^ (95% CI) for post-vaccination		
	Anxiety and nervousness	Mood disorders	Perceptual disturbances and psychoses
Overall	0.95 (0.95–0.96)	0.75 (0.75–0.76)	0.72 (0.70–0.74)
Gender			
Female	0.95 (0.94–0.96)	0.74 (0.73–0.75)	0.72 (0.70–0.75)
Male	0.96 (0.95–0.97)	0.76 (0.75–0.76)	0.72 (0.70–0.75)
Age group			
≤19 years	1.03 (0.97–1.09)	0.98 (0.92–1.04)	0.96 (0.80–1.14)
20–45 years	1.11 (1.09–1.13)	0.81 (0.79–0.83)	0.80 (0.75–0.86)
46–65 years	1.03 (1.02–1.05)	0.87 (0.85–0.88)	0.79 (0.76–0.82)
>65 years	0.75 (0.74–0.76)	0.56 (0.55–0.57)	0.57 (0.55–0.60)
Income level			
First quartile	0.93 (0.91–0.94)	0.74 (0.73–0.76)	0.62 (0.60–0.65)
Second quartile	1.01 (0.99–1.03)	0.81 (0.79–0.83)	0.80 (0.75–0.86)
Third quartile	0.99 (0.97–1.01)	0.80 (0.78–0.82)	0.75 (0.70–0.80)
Fourth quartile	0.92 (0.91–0.93)	0.71 (0.70–0.73)	0.80 (0.76–0.84)
Residential location			
Rural	0.96 (0.95–0.97)	0.76 (0.75–0.77)	0.72 (0.70–0.75)
Metropolitan	0.95 (0.94–0.96)	0.75 (0.74–0.77)	0.72 (0.69–0.75)
Type of platform			
mRNA vaccinations only	0.93 (0.92–0.94)	0.75 (0.74–0.76)	0.72 (0.69–0.75)
Viral vector vaccinations only	1.21 (1.16–1.25)	0.88 (0.84–0.92)	0.72 (0.65–0.80)

	Incidence rate ratio^a^ (95% CI) for post-vaccination		
	Aggression and behavioural disturbances	Cognitive impairments	Sleep disorders
Overall	0.93 (0.89–0.97)	0.68 (0.67–0.69)	0.90 (0.89–0.91)
Gender			
Female	0.97 (0.92–1.03)	0.66 (0.65–0.67)	0.91 (0.89–0.92)
Male	0.89 (0.83–0.94)	0.72 (0.71–0.73)	0.90 (0.88–0.91)
Age group			
≤19 years	0.82 (0.74–0.91)	1.21 (0.85–1.72)	0.98 (0.87–0.10)
20–45 years	0.97 (0.91–1.03)	0.73 (0.71–0.75)	0.96 (0.94–0.99)
46–65 years	0.99 (0.88–1.11)	1.11 (1.08–1.14)	1.05 (1.03–1.06)
>65 years	0.82 (0.73–0.93)	0.61 (0.60–0.62)	0.72 (0.71–0.73)
Income level			
First quartile	0.87 (0.80–0.94)	0.64 (0.62–0.65)	0.88 (0.86–0.90)
Second quartile	0.97 (0.88–1.08)	0.73 (0.71–0.75)	0.96 (0.94–0.99)
Third quartile	0.96 (0.87–1.06)	0.71 (0.69–0.73)	0.94 (0.92–0.96)
Fourth quartile	0.94 (0.87–1.02)	0.68 (0.67–0.69)	0.86 (0.85–0.88)
Residential location			
Rural	0.91 (0.85–0.97)	0.68 (0.63–0.72)	0.89 (0.88–0.91)
Metropolitan	0.96 (0.90–1.02)	0.83 (0.81–0.86)	0.91 (0.90–0.92)
Type of vaccine platform			
mRNA vaccinations only	0.94 (0.89–0.99)	0.79 (0.76–0.82)	0.86 (0.85–0.87)
Viral vector vaccinations only	1.27 (0.99–1.61)	0.93 (0.83–1.03)	1.18 (1.13–1.23)

Pre- and post-vaccination were defined as 21 days after vaccination.

a Season and COVID-19 infections were adjusted using a time-varying approach.

We found consistent results in the sensitivity analyses after excluding cases who died during the study period and those who were infected with COVID-19 at any time during the study period (Supplementary Tables 9–11). According to sensitivity analysis, by splitting the post-vaccination period into three 7-day intervals, all study outcomes showed consistent results with the main findings, except for anxiety and nervousness, and sleep disorders; the risks of those outcomes were slightly increased during the first and third week after COVID-19 vaccination.

## Discussion

In this self-controlled case series study, which included more than 11 million individuals who received the COVID-19 vaccine in South Korea, we observed no association between COVID-19 vaccination and the risk of anxiety and nervousness, mood disorders, perceptual disturbances and psychoses, aggression and behavioural disturbances, cognitive impairments or sleep disorders. However, the risk of anxiety/nervousness or sleep disorders following COVID-19 vaccination increased modestly in individuals with no history of psychiatric disorders. According to stratified analyses, individuals vaccinated with the viral vector only were likely to experience anxiety/nervousness and sleep disorders after vaccination, and an increased risk of anxiety/nervousness, cognitive impairments and sleep disorders was observed in those aged 40–65 years old. Sensitivity analyses showed consistent results with our main findings.

To date, the profile and biological mechanisms underlying the psychiatric manifestations following COVID-19 vaccination remain unclear. Case reports of psychiatric symptoms after COVID-19 vaccination^20–24^ have suggested potential mechanisms involving immune activation through regulation of T-lymphocytes and proinflammatory cytokines.^25^ Cytokines, which are mediators of central nervous system (CNS) activity caused by inflammation resulting from viral infections,^26^ are also involved in modulating neuronal activity in the amygdala, hippocampus, hypothalamus and cerebral cortex.^27^ Proinflammatory cytokines such as interleukin-1β (IL-1β) and tumor necrosis factor-α (TNF-α) have been mostly studied in relation to CNS and are known to be associated with cortical excitability.^28^ IL-1β and TNF-α can be associated not only with seizures, but also with fear/panic attacks and sleep regulation.^29,30^ SARS-CoV-2 infection also shares similar mechanism to psychiatric manifestations and is known to present with a wide range of neuropsychiatric symptoms.^31–33^ A previous study that followed up on COVID-19 survivors for 1 month^34^ showed that 56% of survivors exhibited at least one significant degree of psychopathology. Among these survivors, anxiety and insomnia were the most prevalent symptoms, with rates of 42 and 40%, respectively. However, it remains uncertain whether the risk of developing psychiatric symptoms in patients with COVID-19 is attributed to the viral infection itself, the host’s immune response or the psychological distress related to the diagnosis of COVID-19. Similarly, the association between COVID-19 vaccination and the risk of psychiatric adverse events may also be attributed to the stress caused by vaccination.^35^ During the acute phase of the pandemic, COVID-19 vaccination in South Korea was mandated as a prerequisite for individuals to participate in community activities, suggesting that this situation might have induced psychiatric stress via high pressure on people who do not want to get vaccinated because of fear and uncertainty about the safety of COVID-19 vaccines.

Our study revealed significantly higher psychiatric adverse events in the middle-aged (40–65 years old) group than in the older group. Our findings are not consistent with the hypothesis that an immune response to COVID-19 vaccination can induce psychiatric symptoms through a mechanism similar to SARS-CoV-2 infection, considering that SARS-CoV-2 neurotransmission is known to pose higher risks with advancing age.^36^ Regarding anxiety disorder in this study, the significant increase in the diagnoses following vaccination in the young and middle-aged adult group, in whom such diagnoses are most likely to occur,^37–40^ suggests that vaccination may have triggered latent disorders. Regarding sleep disorders, younger adults are known to exhibit a heightened susceptibility to hypersomnolence, whereas the prevalence of insomnia tends to increase with advancing age.^41,42^ However, the relevant evidence from prior literature reviews is limited; therefore, a more detailed interpretation of our results requires a thorough examination of each specific sleep disorder. In addition, these findings may reflect the unique vulnerabilities of this population during the COVID-19 pandemic, including increased psychosocial stress, occupational instability and caregiving responsibilities, which could have contributed to a higher risk of psychiatric symptoms. The differences from previous studies may be attributable to variations in study populations, settings or analytical methods. Further investigation is warranted to clarify these age-specific patterns and their underlying mechanisms.

It is known that adverse events may vary between different COVID-19 vaccines. For instance, BNT162b2 and mRNA-1273 vaccines have been associated with fewer neurological adverse events than the Ad26.COV2-S vaccine.^43^ In this study, we observed that viral vector vaccines were grossly associated with a higher risk (Supplementary Table 6); however, upon analysing individual vaccines, this trend was not consistent regarding psychiatric adverse events, as these manifestations seemed to differ across each individual vaccine (Supplementary Table 7).

In our study, we observed elevated incidence rates of perceptual disturbances and psychoses during the pre-vaccination period, particularly among individuals with a history of psychiatric disorders. These findings suggest the possibility that symptoms may have emerged before vaccination, or that increased healthcare interactions in anticipation of vaccination might have led to the identification of pre-existing conditions. Although the underlying biological mechanisms remain unclear, this highlights the importance of carefully defining pre-risk periods to detect and account for potential reverse causality in self-controlled case series analyses. Further research is warranted to better understand these patterns and their implications.

It seems that there is no mention of psychiatric-related symptoms and diagnoses related to suspected adverse events after COVID-19 vaccination in South Korea, within the main list of the Korea Centers for Disease Control and Prevention (KCDCP) reported by medical institutions. Additionally, the majority of adverse events reported to the KCDCP were observed within 1 day after vaccination. Anxiety disorder and sleep disorder, which are not common side-effects, might not have been well documented in the Vaccine Adverse Event Reporting Systems in the USA and Europe, unlike our study.^12^ Previously, the Centers for Disease Control had announced that anxiety-related adverse events following immunisation may occur after COVID-19 vaccination. Associated symptoms include tachycardia, hyperventilation, dyspnoea, chest pain, paresthaesia, light-headedness, hypotension, headache, pallor and syncope.^44^ However, it is crucial to note that anxiety-related adverse events following immunisation primarily occurs during the 15-min observation period after vaccination at the vaccination site, and it refers to a condition caused by observing and being influenced by the symptoms of others. This differs significantly from anxiety as an adverse event following vaccination, which is discussed in our study.

To the best of our knowledge, this is the first study to investigate whether COVID-19 vaccination is associated with an increased risk of developing a broad range of psychiatric adverse events. We implemented a self-controlled case series design to address the source of bias from time-invariant confounders by comparing vaccinated and unvaccinated periods within individuals. Our vaccine registry database contained precise information on the timing of vaccination, suggesting a minimal impact of misclassification of the unvaccinated window. In addition, claims data based on a single-payer healthcare system can reflect real-world clinical settings in South Korea. However, this study has several limitations. First, as our study outcomes were assessed based on routinely collected diagnostic codes without additional adjudication, the possibility of outcome misclassification cannot be ruled out, particularly as the data were collected for billing or claims operation, not clinical research. Moreover, the qualifications of assessors might not be available, which could influence the quality of information. However, the validity of diagnostic codes in the claims data has been proven with an 82% predictive positive value when compared with electronic medical records.^45^ Second, a healthy vaccine effect may exist in our study population. However, our self-controlled case series design compared individuals with themselves; thus, we believe that the healthy vaccine was unlikely to affect our findings. Third, despite applying a self-controlled study design that can address time-invariant, unmeasured confounders or residual confounding by time-variant factors may have been present. Fourth, because of the short study period, our findings were largely attributed to the first and second doses, indicating that further studies should be conducted to evaluate the third and fourth doses. Fifth, as our study was based on a single ethnicity, it is unclear whether the findings may be extrapolated or replicated to other ethnic populations. Sixth, other side-effects following vaccination may cause patients to seek medical care in departments other than psychiatry, potentially reducing the likelihood of the visit of psychiatric clinics. However, we conducted a sensitivity analysis by dividing the 21-day risk window into 7-day intervals, and the results showed that the risk in each 1-week period was largely consistent (Supplementary Table 11). This finding suggests that the likelihood of patients visiting psychiatric clinics and having the outcome identified did not differ significantly within our defined risk window. Seventh, the diagnostic codes defined as study outcomes were broadly inclusive. However, certain conditions (such as ICD-10 codes F06, F19, F20 and F91) may raise concerns regarding the temporal link or the need for a longer duration of symptoms than the designated risk period. Therefore, we examined the number of patients diagnosed with ICD-10 codes F06, F19, F20, F91 and F93 from 2021 to 2022, using our database, which includes over 11 million individuals. The results are presented in Supplementary Tables 12 and 13. We found that the proportions of diagnoses of ICD-10 F19, F20, F91 and F93 were very low, ranging from 0.01 to 0.37%. Although the proportion of ICD-10 code F06 was also low at 1.44%, a breakdown of the four-digit codes revealed that most cases (92.1%) were classified as mild cognitive disorder. Additionally, our research team includes a psychiatrist (one of the corresponding authors) with substantial clinical experience treating patients during the COVID-19 pandemic, and their expertise contributed significantly to the definition of the study outcomes. Therefore, we believe that these diagnostic codes did not have a significant impact on our findings. Lastly, our study population included individuals with pre-existing psychiatric conditions, which introduces the potential for bias caused by concurrent use of psychotropic medications that was not adjusted for in the self-controlled case series model. Although this source of bias cannot be completely ruled out, psychotropic medications prescribed for psychiatric conditions are typically administered on a daily, long-term basis. Therefore, we believe that the self-controlled case series study design might minimise potential bias arising from this factor. However, since some psychotropic medications are intended for short-term use, cautious interpretation is needed. Future studies should consider these medications as potential confounding factors or clarify their potential impact on study outcomes.

In conclusion, we did not find an association between COVID-19 vaccines and the short-term risk of anxiety and nervousness, mood disorders, perceptual disturbances and psychoses, aggression and behavioural disturbances, cognitive impairments or sleep disorders. However, we found a modestly increased risk of anxiety/nervousness or sleep disorders following COVID-19 vaccination in individuals without any history of psychiatric disorders. As we wait for the results of confirmatory studies, our findings provide short-term safety profiles for COVID-19 vaccines regarding psychiatric adverse events, and support that individuals with psychiatric disorders should be included in the priority groups for vaccination. In the meantime, continuous monitoring of anxiety/nervousness or sleep disorders after COVID-19 vaccination is required, even when vaccinated individuals have no history of psychiatric comorbidities.

## Supporting information

Lee et al. supplementary material Lee et al. supplementary material

## Data Availability

The data that support the findings of this study are available from the National Health Insurance Service of South Korea, but restrictions apply to the availability of these data due to domestic laws and regulations that prohibit the distribution or release of individuals’ data to the public and so are not publicly available. Data are, however, available from the authors on reasonable request and with permission of National Health Insurance Service of South Korea.

## Supplementary material

The supplementary material is available online at https://doi.org/10.1192/bjo.2025.10803
