# Supplementary material for: Short-term risk of psychiatric adverse events following COVID-19 vaccination: nationwide self-controlled case series study
Source: BJPsych Open. 2025 Sep 22;11(5):e218. doi: 10.1192/bjo.2025.10803 (PMC12458088; doi:10.1192/bjo.2025.10803)
Supplement: Lee et al. supplementary material [file S205647242510803Xsup001.docx]

**Supplementary materials**

**Supplementary figure 1.** Graphical depiction of self-controlled case series design

**Supplementary table 1**. Operational definition of neuropsychiatric adverse events

**Supplementary table 2.** Risk of psychiatric adverse events following COVID-19 vaccination in South Korea, stratified by sex

**Supplementary table 3.** Risk of psychiatric adverse events following COVID-19 vaccination in South Korea, stratified by age group

**Supplementary table 4.** Risk of psychiatric adverse events following COVID-19 vaccination in South Korea, stratified by income level

**Supplementary table 5.** Risk of psychiatric adverse events following COVID-19 vaccination in South Korea, stratified by residential location

**Supplementary table 6.** Risk of psychiatric adverse events following COVID-19 vaccination in South Korea, stratified by the type of vaccine platform

**Supplementary table 7.** Risk of psychiatric adverse events following COVID-19 vaccination in South Korea, stratified by individual vaccines

**Supplementary table 8.** Risk of psychiatric adverse events following COVID-19 vaccination in South Korea, stratified by vaccination dose

**Supplementary table 9.** Sensitivity analyses for the Risk of psychiatric adverse events following COVID-19 vaccination in South Korea: Excluding death cases during the study period

**Supplementary table 10.** Sensitivity analyses for the Risk of psychiatric adverse events following COVID-19 vaccination in South Korea: Excluding COVID-19 cases during the study period

**Supplementary table 11.** Sensitivity analyses for the Risk of psychiatric adverse events following COVID-19 vaccination in South Korea: Splitting the risk interval

**Supplementary table 12.** The number of patients diagnosed with diagnostic code in the total study cohort of our study

**Supplementary table 13.** The number of patients according to 4-digit ICD-10 codes among those diagnosed with F06

**
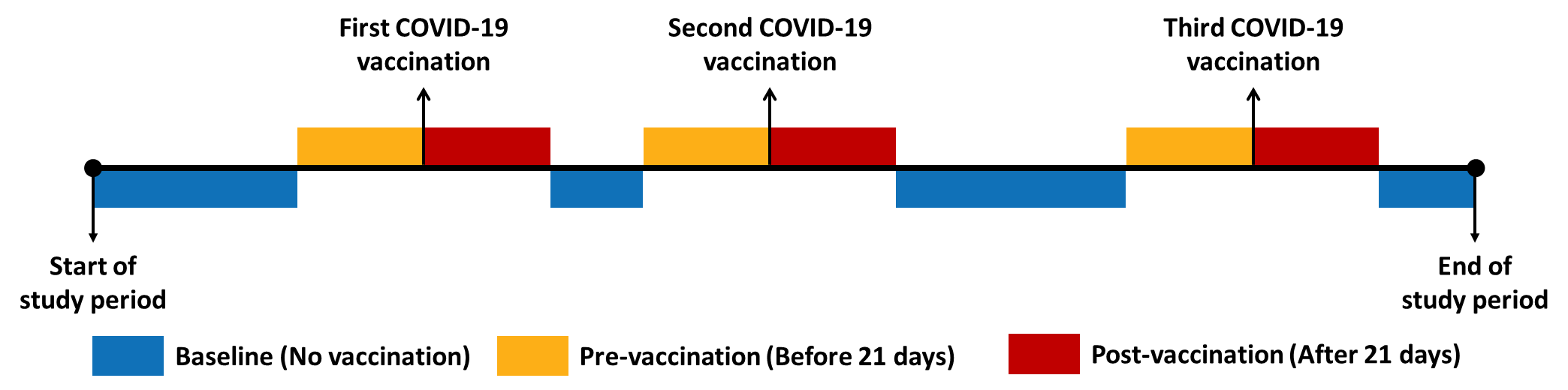
**

**Supplementary figure 1.** Graphical depiction of the self-controlled case series study design

**Supplementary table 1.** Operational definition of neuropsychiatric adverse events

| **Type of outcomes** | **Diagnostic code (ICD-10)** |
| --- | --- |
| Anxiety and nervousness | F064, F40, F41, F43, F45, F48, R450, R451, R457 |
| Mood disorders | F063, F066, F30, F31, F32, F33, F34, F38, F39, F53, R452, R453 |
| Perceptual disturbances  and psychoses | F060, F061, F062, F09, F20, F21, F22, F23, F24, F25, F28, F29, H932, R44 |
| Aggression and  behavioral disturbances | F19, F90, F91, F92, F93, F94, F95, F96, F97, F98, R454, R455, R456, R458, X60, X61, X62, X63, X64, X65, X66, X67, X68, X69, X70, X71, X72, X73, X74, X75, X76, X77, X78, X79, X80, X81, X82, X83, X84 |
| Cognitive impairments | F00, F01, F02, F03, F04, F05, F065, F067, F068, F069, F07, F09, R40, R41 |
| Sleep disorder | F51, G47 |

**Abbreviations**: ICD-10, International Classification of Diseases 10th revision.

**Supplementary table 2.** Risk of psychiatric adverse events following COVID-19 vaccination in South Korea, stratified by sex

|  | **Female** | | | **Male** | | |
| --- | --- | --- | --- | --- | --- | --- |
|  | **No. of**  **events** | **Incidence rate^*^**  **(95% CI)** | **Incidence rate ratio^†^**  **(95% CI)** | **No. of**  **events** | **Incidence rate^*^**  **(95% CI)** | **Incidence rate ratio^†^**  **(95% CI)** |
| **Anxiety and nervousness** | |  |  |  |  |  |
| Baseline | 268,557 | 3.57 (3.56 to 3.58) | 1.00 (Reference) | 161,349 | 3.60 (3.58 to 3.61) | 1.00 (Reference) |
| Pre-vaccination | 35,085 | 2.39 (2.37 to 2.42) | 0.83 (0.82 to 0.84) | 19,364 | 2.28 (2.24 to 2.31) | 0.81 (0.80 to 0.82) |
| Post-vaccination | 38,675 | 2.43 (2.41 to 2.46) | 0.95 (0.94 to 0.96) | 21,753 | 2.37 (2.34 to 2.40) | 0.96 (0.95 to 0.97) |
| **Mood disorders** |  |  |  |  |  |  |
| Baseline | 243,033 | 3.76 (3.75 to 3.78) | 1.00 (Reference) | 381,345 | 3.75 (3.74 to 3.76) | 1.00 (Reference) |
| Pre-vaccination | 25,012 | 2.05 (2.03 to 2.08) | 0.74 (0.73 to 0.75) | 39,797 | 2.09 (2.07 to 2.11) | 0.75 (0.75 to 0.76) |
| Post-vaccination | 23,797 | 1.77 (1.75 to 1.79) | 0.74 (0.73 to 0.75) | 37,207 | 1.78 (1.76 to 1.80) | 0.76 (0.75 to 0.76) |
| **Perceptual disturbances and psychoses** | |  |  |  |  |  |
| Baseline | 39,889 | 3.65 (3.62 to 3.69) | 1.00 (Reference) | 32,272 | 3.51 (3.47 to 3.55) | 1.00 (Reference) |
| Pre-vaccination | 5,583 | 2.79 (2.72 to 2.86) | 1.10 (1.07 to 1.13) | 5,981 | 3.59 (3.50 to 3.68) | 1.44 (1.40 to 1.47) |
| Post-vaccination | 3,357 | 1.55 (1.50 to 1.60) | 0.72 (0.70 to 0.75) | 2,800 | 1.54 (1.48 to 1.59) | 0.72 (0.70 to 0.75) |
| **Aggression and behavioral disturbances** | |  |  |  |  |  |
| Baseline | 11,150 | 3.51 (3.45 to 3.58) | 1.00 (Reference) | 15,553 | 3.68 (3.62 to 3.74) | 1.00 (Reference) |
| Pre-vaccination | 1,143 | 2.45 (2.32 to 2.60) | 0.97 (0.91 to 1.03) | 883 | 1.68 (1.57 to 1.80) | 0.82 (0.77 to 0.88) |
| Post-vaccination | 1,282 | 2.31 (2.19 to 2.44) | 0.97 (0.92 to 1.03) | 1,064 | 1.62 (1.53 to 1.73) | 0.89 (0.83 to 0.94) |
| **Cognitive impairments** |  |  |  |  |  |  |
| Baseline | 181,870 | 3.58 (3.56 to 3.60) | 1.00 (Reference) | 85,136 | 3.52 (3.50 to 3.55) | 1.00 (Reference) |
| Pre-vaccination | 28,977 | 2.80 (2.77 to 2.84) | 0.73 (0.72 to 0.73) | 13,958 | 2.92 (2.87 to 2.97) | 0.78 (0.77 to 0.80) |
| Post-vaccination | 25,790 | 2.15 (2.13 to 2.18) | 0.66 (0.65 to 0.67) | 12,702 | 2.27 (2.23 to 2.31) | 0.72 (0.71 to 0.73) |
| **Sleep disorder** |  |  |  |  |  |  |
| Baseline | 203,268 | 3.66 (3.64 to 3.67) | 1.00 (Reference) | 140,172 | 3.65 (3.64 to 3.67) | 1.00 (Reference) |
| Pre-vaccination | 25,625 | 2.22 (2.19 to 2.24) | 0.76 (0.75 to 0.77) | 17,760 | 2.26 (2.23 to 2.29) | 0.77 (0.76 to 0.78) |
| Post-vaccination | 28,330 | 2.30 (2.27 to 2.32) | 0.91 (0.89 to 0.92) | 18,851 | 2.24 (2.21 to 2.27) | 0.90 (0.88 to 0.91) |

**Abbreviations**: CI, confidence interval.

Pre-vaccination and post-vaccination were defined as 21 days before and after the vaccination date, respectively.

^*^Incidence rate was calculated per 1,000 person-days.

^†^Season and infection of COVID-19 were adjusted with a time-varying approach.

**Supplementary table 3.** Risk of psychiatric adverse events following COVID-19 vaccination in South Korea, stratified by age group

|  | **No. of**  **events** | **Incidence rate^*^**  **(95% CI)** | **Incidence rate ratio^†^**  **(95% CI)** |
| --- | --- | --- | --- |
| **≤ 19 years old** | |  |  |
| **Anxiety and nervousness** | |  |  |
| Baseline | 13,414 | 3.39 (3.34 to 3.45) | 1.00 (Reference) |
| Pre-vaccination | 981 | 2.45 (2.30 to 2.61) | 0.92 (0.86 to 0.99) |
| Post-vaccination | 1,618 | 2.72 (2.59 to 2.85) | 1.03 (0.97 to 1.09) |
| **Mood disorders** |  |  |  |
| Baseline | 14,552 | 3.50 (3.44 to 3.56) | 1.00 (Reference) |
| Pre-vaccination | 897 | 2.14 (2.01 to 2.29) | 0.95 (0.88 to 1.02) |
| Post-vaccination | 1,377 | 2.21 (2.09 to 2.33) | 0.98 (0.92 to 1.04) |
| **Perceptual disturbances and psychoses** | |  |  |
| Baseline | 1,711 | 3.52 (3.36 to 3.69) | 1.00 (Reference) |
| Pre-vaccination | 89 | 1.95 (1.56 to 2.37) | 0.87 (0.70 to 1.09) |
| Post-vaccination | 146 | 2.12 (1.80 to 2.50) | 0.96 (0.80 to 1.14) |
| **Aggression and behavioral disturbances** | |  |  |
| Baseline | 9,354 | 3.63 (3.56 to 3.70) | 1.00 (Reference) |
| Pre-vaccination | 265 | 1.29 (1.14 to 1.46) | 0.86 (0.76 to 0.98) |
| Post-vaccination | 441 | 1.35 (1.23 to 1.49) | 0.82 (0.74 to 0.91) |
| **Cognitive impairments** |  |  |  |
| Baseline | 323 | 3.33 (2.99 to 3.72) | 1.00 (Reference) |
| Pre-vaccination | 21 | 2.55 (1.66 to 3.91) | 1.01 (0.64 to 1.58) |
| Post-vaccination | 36 | 2.92 (2.11 to 4.05) | 1.21 (0.85 to 1.72) |
| **Sleep disorders** |  |  |  |
| Baseline | 2,710 | 3.37 (3.25 to 3.50) | 1.00 (Reference) |
| Pre-vaccination | 239 | 2.66 (2.34 to 3.02) | 0.93 (0.82 to 1.07) |
| Post-vaccination | 355 | 2.78 (2.51 to 3.09) | 0.98 (0.87 to 0.10) |
| **20-45 years old** | |  |  |
| **Anxiety and nervousness** | |  |  |
| Baseline | 133,712 | 3.49 (3.47 to 3.51) | 1.00 (Reference) |
| Pre-vaccination | 12,546 | 2.24 (2.20 to 2.28) | 0.91 (0.90 to 0.93) |
| Post-vaccination | 16,578 | 2.58 (2.54 to 2.62) | 1.11 (1.09 to 1.13) |
| **Mood disorders** |  |  |  |
| Baseline | 130,168 | 3.66 (3.64 to 3.68) | 1.00 (Reference) |
| Pre-vaccination | 10,524 | 2.00 (1.96 to 2.04) | 0.77 (0.75 to 0.79) |
| Post-vaccination | 11,202 | 1.85 (1.81 to 1.88) | 0.81 (0.79 to 0.83) |
| **Perceptual disturbances and psychoses** | |  |  |
| Baseline | 23,856 | 3.68 (3.63 to 3.72) | 1.00 (Reference) |
| Pre-vaccination | 2,384 | 2.47 (2.37 to 2.57) | 0.97 (0.92 to 1.03) |
| Post-vaccination | 1,469 | 1.33 (1.26 to 1.40) | 0.80 (0.75 to 0.86) |
| **Aggression and behavioral disturbances** | |  |  |
| Baseline | 13,289 | 3.63 (3.57 to 3.70) | 1.00 (Reference) |
| Pre-vaccination | 1,074 | 1.99 (1.87 to 3.70) | 0.91 (0.86 to 0.97) |
| Post-vaccination | 1,238 | 1.98 (1.88 to 2.10) | 0.97 (0.91 to 1.03) |
| **Cognitive impairments** |  |  |  |
| Baseline | 3,208 | 3.45 (3.34 to 3.58) | 1.00 (Reference) |
| Pre-vaccination | 390 | 2.81 (2.55 to 3.11) | 0.74 (0.72 to 0.76) |
| Post-vaccination | 362 | 2.31 (2.09 to 2.57) | 0.73 (0.71 to 0.75) |
| **Sleep disorders** |  |  |  |
| Baseline | 79,268 | 3.55 (3.52 to 3.57) | 1.00 (Reference) |
| Pre-vaccination | 7,482 | 2.21 (2.16 to 2.26) | 0.78 (0.76 to 0.79) |
| Post-vaccination | 9,028 | 2.34 (2.29 to 2.39) | 0.96 (0.94 to 0.99) |
| **46-65 years old** | |  |  |
| **Anxiety and nervousness** | |  |  |
| Baseline | 154,592 | 3.64 (3.62 to 3.66) | 1.00 (Reference) |
| Pre-vaccination | 20,368 | 2.22 (2.19 to 2.25) | 0.86 (0.84 to 0.87) |
| Post-vaccination | 21,000 | 2.36 (2.33 to 2.39) | 1.03 (1.02 to 1.05) |
| **Mood disorders** |  |  |  |
| Baseline | 120,741 | 3.85 (3.83 to 3.87) | 1.00 (Reference) |
| Pre-vaccination | 12,701 | 1.90 (1.87 to 1.94) | 0.82 (0.81 to 0.84) |
| Post-vaccination | 10,787 | 1.65 (1.62 to 1.68) | 0.87 (0.85 to 0.88) |
| **Perceptual disturbances and psychoses** | |  |  |
| Baseline | 29,953 | 3.47 (3.43 to 3.51) | 1.00 (Reference) |
| Pre-vaccination | 6,642 | 3.90 (3.81 to 3.99) | 1.85 (1.81 to 1.89) |
| Post-vaccination | 2,567 | 1.46 (1.40 to 1.52) | 0.79 (0.76 to 0.82) |
| **Aggression and behavioral disturbances** | |  |  |
| Baseline | 2,350 | 3.53 (3.39 to 3.67) | 1.00 (Reference) |
| Pre-vaccination | 351 | 2.55 (2.30 to 2.83) | 0.93 (0.84 to 1.05) |
| Post-vaccination | 343 | 2.51 (2.26 to 2.79) | 0.99 (0.88 to 1.11) |
| **Cognitive impairments** |  |  |  |
| Baseline | 34,354 | 3.49 (3.45 to 3.52) | 1.00 (Reference) |
| Pre-vaccination | 6,544 | 2.79 (2.73 to 2.86) | 1.03 (1.00 to 1.06) |
| Post-vaccination | 5,860 | 2.60 (2.54 to 2.67) | 1.11 (1.08 to 1.14) |
| **Sleep disorders** |  |  |  |
| Baseline | 138,179 | 3.69 (3.67 to 3.71) | 1.00 (Reference) |
| Pre-vaccination | 17,605 | 2.10 (2.07 to 2.13) | 0.82 (0.81 to 0.84) |
| Post-vaccination | 18,720 | 2.31 (2.27 to 2.34) | 1.05 (1.03 to 1.06) |
| **>65 years old** | |  |  |
| **Anxiety and nervousness** | |  |  |
| Baseline | 128,188 | 3.63 (3.61 to 3.65) | 1.00 (Reference) |
| Pre-vaccination | 20,554 | 2.56 (2.53 to 2.60) | 0.69 (0.68 to 0.70) |
| Post-vaccination | 21,232 | 2.32 (2.29 to 2.35) | 0.75 (0.74 to 0.76) |
| **Mood disorders** |  |  |  |
| Baseline | 115,884 | 3.79 (3.77 to 3.81) | 1.00 (Reference) |
| Pre-vaccination | 15,645 | 2.35 (2.32 to 2.39) | 0.58 (0.57 to 0.59) |
| Post-vaccination | 13,841 | 1.80 (1.77 to 1.83) | 0.56 (0.55 to 0.57) |
| **Perceptual disturbances and psychoses** | |  |  |
| Baseline | 16,641 | 3.70 (3.64 to 3.75) | 1.00 (Reference) |
| Pre-vaccination | 2,449 | 2.58 (2.48 to 2.68) | 0.65 (0.62 to 0.67) |
| Post-vaccination | 1,975 | 1.87 (1.79 to 1.95) | 0.57 (0.55 to 0.60) |
| **Aggression and behavioral disturbances** | |  |  |
| Baseline | 1,710 | 3.14 (3.26 to 3.58) | 1.00 (Reference) |
| Pre-vaccination | 336 | 3.11 (2.79 to 3.46) | 0.88 (0.79 to 0.99) |
| Post-vaccination | 324 | 2.63 (2.36 to 2.93) | 0.82 (0.73 to 0.93) |
| **Cognitive impairments** |  |  |  |
| Baseline | 229,121 | 3.57 (0.56 to 3.59) | 1.00 (Reference) |
| Pre-vaccination | 35,980 | 2.85 (2.82 to 2.88) | 0.68 (0.67 to 0.69) |
| Post-vaccination | 32,234 | 2.13 (2.10 to 2.15) | 0.61 (0.60 to 0.62) |
| **Sleep disorders** |  |  |  |
| Baseline | 123,283 | 3.70 (3.68 to 3.72) | 1.00 (Reference) |
| Pre-vaccination | 18,059 | 2.39 (2.35 to 2.42) | 0.63 (0.62 to 0.64) |
| Post-vaccination | 19,078 | 2.21 (2.18 to 2.24) | 0.72 (0.71 to 0.73) |

**Abbreviations**: CI, confidence interval.

Pre-vaccination and post-vaccination were defined as 21 days before and after the vaccination date, respectively.

^*^Incidence rate was calculated per 1,000 person-days.

^†^Season and infection of COVID-19 were adjusted with a time-varying approach.

**Supplementary table 4.** Risk of psychiatric adverse events following COVID-19 vaccination in South Korea, stratified by income level

|  | **No. of**  **events** | **Incidence rate^*^**  **(95% CI)** | **Incidence rate ratio^†^**  **(95% CI)** |
| --- | --- | --- | --- |
| **1^st^ quartile (Most deprived)** | |  |  |
| **Anxiety and nervousness** | |  |  |
| Baseline | 117,282 | 3.60 (3.58 to 3.62) | 1.00 (Reference) |
| Pre-vaccination | 14,687 | 2.35 (2.31 to 2.39) | 0.83 (0.82 to 0.85) |
| Post-vaccination | 15,389 | 2.30 (2.26 to 2.34) | 0.93 (0.91 to 0.94) |
| **Mood disorders** |  |  |  |
| Baseline | 119,048 | 3.75 (3.73 to 3.77) | 1.00 (Reference) |
| Pre-vaccination | 12,951 | 2.18 (2.15 to 2.22) | 0.82 (0.80 to 0.83) |
| Post-vaccination | 10,733 | 1.67 (1.64 to 1.70) | 0.74 (0.73 to 0.76) |
| **Perceptual disturbances and psychoses** | |  |  |
| Baseline | 33,755 | 3.45 (3.41 to 3.49) | 1.00 (Reference) |
| Pre-vaccination | 7,495 | 4.09 (4.00 to 4.19) | 1.60 (1.57 to 1.64) |
| Post-vaccination | 2,757 | 1.39 (1.34 to 1.44) | 0.62 (0.60 to 0.65) |
| **Aggression and behavioral disturbances** | |  |  |
| Baseline | 7,422 | 3.64 (3.56 to 3.72) | 1.00 (Reference) |
| Pre-vaccination | 568 | 2.04 (1.88 to 2.21) | 0.90 (0.83 to 0.98) |
| Post-vaccination | 612 | 1.81 (1.67 to 1.96) | 0.87 (0.80 to 0.94) |
| **Cognitive impairments** |  |  |  |
| Baseline | 85,272 | 3.57 (3.55 to 3.59) | 1.00 (Reference) |
| Pre-vaccination | 13,820 | 2.91 (2.86 to 0.30) | 0.76 (0.75 to 0.78) |
| Post-vaccination | 10,980 | 2.04 (2.00 to 2.08) | 0.64 (0.62 to 0.65) |
| **Sleep disorders** |  |  |  |
| Baseline | 99,290 | 3.67 (3.65 to 3.70) | 1.00 (Reference) |
| Pre-vaccination | 12,330 | 2.24 (2.20 to 2.28) | 0.78 (0.76 to 0.79) |
| Post-vaccination | 12,559 | 2.16 (2.12 to 2.19) | 0.88 (0.86 to 0.90) |
| **2^nd^ quartile** | |  |  |
| **Anxiety and nervousness** | |  |  |
| Baseline | 81,529 | 3.55 (3.53 to 3.58) | 1.00 (Reference) |
| Pre-vaccination | 10,023 | 2.32 (2.28 to 2.37) | 0.83 (0.82 to 0.85) |
| Post-vaccination | 11,697 | 2.52 (2.48 to 2.57) | 0.93 (0.91 to 0.94) |
| **Mood disorders** |  |  |  |
| Baseline | 69,659 | 3.73 (3.70 to 3.76) | 1.00 (Reference) |
| Pre-vaccination | 6,904 | 2.04 (1.99 to 2.08) | 0.77 (0.75 to 0.79) |
| Post-vaccination | 6,866 | 1.85 (1.81 to 1.90) | 0.81 (0.79 to 0.83) |
| **Perceptual disturbances and psychoses** | |  |  |
| Baseline | 10,876 | 3.72 (3.65 to 3.79) | 1.00 (Reference) |
| Pre-vaccination | 1,147 | 2.27 (2.14 to 2.41) | 0.97 (0.92 to 1.03) |
| Post-vaccination | 856 | 1.56 (1.46 to 1.67) | 0.80 (0.75 to 0.86) |
| **Aggression and behavioral disturbances** | |  |  |
| Baseline | 4,893 | 3.57 (3.48 to 3.68) | 1.00 (Reference) |
| Pre-vaccination | 432 | 2.23 (2.03 to 2.45) | 0.97 (0.88 to 1.07) |
| Post-vaccination | 472 | 2.06 (1.88 to 2.25) | 0.97 (0.88 to 1.08) |
| **Cognitive impairments** |  |  |  |
| Baseline | 37,614 | 3.56 (3.53 to 3.60) | 1.00 (Reference) |
| Pre-vaccination | 5,845 | 2.73 (2.66 to 2.80) | 0.74 (0.72 to 0.76) |
| Post-vaccination | 5,522 | 2.26 (2.20 to 2.32) | 0.73 (0.71 to 0.75) |
| **Sleep disorders** |  |  |  |
| Baseline | 63,406 | 3.64 (3.61 to 3.67) | 1.00 (Reference) |
| Pre-vaccination | 7,626 | 2.17 (2.12 to 2.22) | 0.78 (0.76 to 0.79) |
| Post-vaccination | 8,722 | 2.35 (2.30 to 2.40) | 0.96 (0.94 to 0.99) |
| **3^rd^ quartile** | |  |  |
| **Anxiety and nervousness** | |  |  |
| Baseline | 90,723 | 3.57 (3.55 to 3.59) | 1.00 (Reference) |
| Pre-vaccination | 11,471 | 2.34 (2.29 to 2.38) | 0.83 (0.81 to 0.84) |
| Post-vaccination | 12,955 | 2.47 (2.42 to 2.51) | 0.99 (0.97 to 1.01) |
| **Mood disorders** |  |  |  |
| Baseline | 74,692 | 3.75 (3.72 to 3.77) | 1.00 (Reference) |
| Pre-vaccination | 7,484 | 2.01 (1.96 to 2.05) | 0.73 (0.72 to 0.75) |
| Post-vaccination | 7,538 | 1.86 (1.81 to 1.90) | 0.80 (0.78 to 0.82) |
| **Perceptual disturbances and psychoses** | |  |  |
| Baseline | 11,311 | 3.77 (3.70 to 3.84) | 1.00 (Reference) |
| Pre-vaccination | 1,109 | 2.07 (1.95 to 2.19) | 0.83 (0.78 to 0.88) |
| Post-vaccination | 891 | 1.54 (1.44 to 1.64) | 0.75 (0.70 to 0.80) |
| **Aggression and behavioral disturbances** | |  |  |
| Baseline | 5,112 | 3.60 (3.50 to 3.70) | 1.00 (Reference) |
| Pre-vaccination | 380 | 1.97 (1.78 to 2.17) | 0.83 (0.75 to 0.92) |
| Post-vaccination | 483 | 2.07 (1.89 to 2.26) | 0.96 (0.87 to 1.06) |
| **Cognitive impairments** |  |  |  |
| Baseline | 47,072 | 3.58 (3.55 to 3.61) | 1.00 (Reference) |
| Pre-vaccination | 7,402 | 2.74 (2.67 to 2.80) | 0.74 (0.72 to 0.76) |
| Post-vaccination | 6,810 | 2.20 (2.15 to 2.26) | 0.71 (0.69 to 0.73) |
| **Sleep disorders** |  |  |  |
| Baseline | 69,342 | 3.64 (3.62 to 3.67) | 1.00 (Reference) |
| Pre-vaccination | 8,741 | 2.22 (2.17 to 2.27) | 0.77 (0.76 to 0.79) |
| Post-vaccination | 9,667 | 2.33 (2.28 to 2.37) | 0.94 (0.92 to 0.96) |
| **4^th^ quartile (Most affluent)** | |  |  |
| **Anxiety and nervousness** | |  |  |
| Baseline | 140,372 | 3.59 (3.57 to 3.61) | 1.00 (Reference) |
| Pre-vaccination | 18,268 | 2.37 (2.34 to 2.41) | 0.80 (0.79 to 0.81) |
| Post-vaccination | 20,387 | 2.40 (2.37 to 2.43) | 0.92 (0.91 to 0.93) |
| **Mood disorders** |  |  |  |
| Baseline | 117,946 | 3.76 (3.74 to 3.78) | 1.00 (Reference) |
| Pre-vaccination | 12,428 | 2.09 (2.05 to 2.13) | 0.71 (0.69 to 0.72) |
| Post-vaccination | 12,070 | 1.80 (1.77 to 1.83) | 0.71 (0.70 to 0.73) |
| **Perceptual disturbances and psychoses** | |  |  |
| Baseline | 16,219 | 3.68 (3.62 to 3.73) | 1.00 (Reference) |
| Pre-vaccination | 1,813 | 2.29 (2.18 to 2.39) | 0.83 (0.79 to 0.87) |
| Post-vaccination | 1,653 | 1.88 (1.79 to 1.97) | 0.80 (0.76 to 0.84) |
| **Aggression and behavioral disturbances** | |  |  |
| Baseline | 9,276 | 3.61 (3.53 to 3.68) | 1.00 (Reference) |
| Pre-vaccination | 646 | 1.99 (1.84 to 2.15) | 0.90 (0.83 to 0.97) |
| Post-vaccination | 779 | 1.91 (1.78 to 2.05) | 0.94 (0.87 to 1.02) |
| **Cognitive impairments** |  |  |  |
| Baseline | 97,048 | 3.54 (3.52 to 3.56) | 1.00 (Reference) |
| Pre-vaccination | 15,868 | 2.87 (2.83 to 2.92) | 0.73 (0.72 to 0.74) |
| Post-vaccination | 15,180 | 2.28 (2.25 to 2.32) | 0.68 (0.67 to 0.69) |
| **Sleep disorders** |  |  |  |
| Baseline | 111,402 | 3.66 (3.64 to 3.68) | 1.00 (Reference) |
| Pre-vaccination | 14,688 | 2.27 (2.24 to 2.31) | 0.74 (0.72 to 0.75) |
| Post-vaccination | 16,233 | 2.30 (2.26 to 2.33) | 0.86 (0.85 to 0.88) |

**Abbreviations**: CI, confidence interval.

Pre-vaccination and post-vaccination were defined as 21 days before and after the vaccination date, respectively.

^*^Incidence rate was calculated per 1,000 person-days.

^†^Season and infection of COVID-19 were adjusted with a time-varying approach.

**Supplementary table 5.** Risk of psychiatric adverse events following COVID-19 vaccination in South Korea, stratified by residential location

|  | **Rural** | | | **Metropolitan^*^** | | |
| --- | --- | --- | --- | --- | --- | --- |
|  | **No. of**  **events** | **Incidence rate^†^**  **(95% CI)** | **Incidence rate ratio^‡^**  **(95% CI)** | **No. of**  **events** | **Incidence rate^†^**  **(95% CI)** | **Incidence rate ratio^‡^**  **(95% CI)** |
| **Anxiety and nervousness** | |  |  |  |  |  |
| Baseline | 238,999 | 3.57 (3.55 to 3.58) | 1.00 (Reference) | 190,907 | 3.60 (3.58 to 3.61) | 1.00 (Reference) |
| Pre-vaccination | 31,418 | 2.39 (2.37 to 2.42) | 0.83 (0.82 to 0.84) | 23,031 | 2.29 (2.26 to 2.32) | 0.82 (0.80 to 0.83) |
| Post-vaccination | 35,051 | 2.46 (2.44 to 2.49) | 0.96 (0.95 to 0.97) | 25,377 | 2.34 (2.31 to 2.37) | 0.95 (0.94 to 0.96) |
| **Mood disorders** |  |  |  |  |  |  |
| Baseline | 208,772 | 3.73 (3.72 to 3.75) | 1.00 (Reference) | 172,573 | 3.77 (3.75 to 3.79) | 1.00 (Reference) |
| Pre-vaccination | 22,951 | 2.16 (2.13 to 2.19) | 0.76 (0.75 to 0.77) | 16,816 | 2.01 (1.98 to 2.04) | 0.75 (0.73 to 0.76) |
| Post-vaccination | 21,528 | 1.84 (1.81 to 1.86) | 0.76 (0.75 to 0.77) | 15,679 | 1.71 (1.68 to 1.73) | 0.75 (0.74 to 0.77) |
| **Perceptual disturbances and psychoses** | |  |  |  |  |  |
| Baseline | 39,597 | 3.55 (3.52 to 3.59) | 1.00 (Reference) | 32,564 | 3.63 (0.36 to 3.67) | 1.00 (Reference) |
| Pre-vaccination | 6,847 | 3.31 (3.23 to 3.38) | 1.28 (1.25 to 1.31) | 4,717 | 2.96 (2.87 to 3.04) | 0.90 (0.88 to 0.93) |
| Post-vaccination | 3,656 | 1.62 (1.56 to 1.67) | 0.72 (0.70 to 0.75) | 2,501 | 1.45 (1.39 to 1.50) | 0.72 (0.69 to 0.75) |
| **Aggression and behavioral disturbances** | |  |  |  |  |  |
| Baseline | 12,843 | 3.60 (3.54 to 3.66) | 1.00 (Reference) | 13,860 | 3.62 (3.65 to 3.68) | 1.00 (Reference) |
| Pre-vaccination | 1,012 | 2.11 (1.99 to 2.25) | 0.92 (0.86 to 0.98) | 1,014 | 1.98 (1.86 to 2.11) | 0.89 (0.83 to 0.95) |
| Post-vaccination | 1,137 | 1.94 (1.83 to 2.06) | 0.91 (0.85 to 0.97) | 1,209 | 1.94 (1.83 to 2.05) | 0.96 (0.90 to 1.02) |
| **Cognitive impairments** |  |  |  |  |  |  |
| Baseline | 163,037 | 3.56 (3.55 to 3.58) | 1.00 (Reference) | 103,969 | 3.56 (3.54 to 3.58) | 1.00 (Reference) |
| Pre-vaccination | 26,334 | 2.85 (2.82 to 2.89) | 0.73 (0.72 to 0.74) | 16,601 | 2.82 (2.78 to 2.86) | 0.74 (0.73 to 0.75) |
| Post-vaccination | 23,597 | 2.18 (2.15 to 2.21) | 0.65 (0.64 to 0.66) | 14,895 | 2.20 (2.17 to 2.24) | 0.69 (0.68 to 0.70) |
| **Sleep disorder** |  |  |  |  |  |  |
| Baseline | 183,789 | 3.65 (3.63 to 3.64) | 1.00 (Reference) | 159,651 | 3.66 (3.64 to 3.68) | 1.00 (Reference) |
| Pre-vaccination | 23,600 | 2.25 (2.22 to 2.28) | 0.76 (0.75 to 0.77) | 19,785 | 2.21 (2.18 to 2.24) | 0.76 (0.75 to 0.77) |
| Post-vaccination | 25,734 | 2.30 (2.27 to 2.32) | 0.89 (0.88 to 0.91) | 21,447 | 2.25 (2.22 to 2.28) | 0.91 (0.90 to 0.92) |

**Abbreviations**: CI, confidence interval.

Pre-vaccination and post-vaccination were defined as 21 days before and after the vaccination date, respectively.

^*^ metropolitan area includes 10 large cities with a population of over one million.

^†^ incidence rate was calculated per 1,000 person-days.

^‡^ Season and infection of COVID-19 were adjusted with a time-varying approach.

**Supplementary table 6.** Risk of psychiatric adverse events following COVID-19 vaccination in South Korea, stratified by the type of platform

|  | **mRNA vaccinations only^*^** | | | **Viral vector vaccinations only^*^** | | |
| --- | --- | --- | --- | --- | --- | --- |
|  | **No. of**  **events** | **Incidence rate^†^**  **(95% CI)** | **Incidence rate ratio^‡^**  **(95% CI)** | **No. of**  **events** | **Incidence rate^†^**  **(95% CI)** | **Incidence rate ratio^‡^**  **(95% CI)** |
| **Anxiety and nervousness** | |  |  |  |  |  |
| Baseline | 298,224 | 3.54 (3.52 to 3.55) | 1.00 (Reference) | 56,994 | 3.31 (3.29 to 3.34) | 1.00 (Reference) |
| Pre-vaccination | 29,947 | 2.31 (2.29 to 2.34) | 0.88 (0.87 to 0.89) | 2,648 | 2.50 (2.41 to 2.60) | 0.82 (0.79 to 0.85) |
| Post-vaccination | 36,925 | 2.37 (2.35 to 2.40) | 0.93 (0.92 to 0.94) | 2,904 | 2.72 (2.63 to 2.83) | 1.21 (1.16 to 1.25) |
| **Mood disorders** |  |  |  |  |  |  |
| Baseline | 270,362 | 3.69 (3.68 to 3.71) | 1.00 (Reference) | 51,922 | 3.36 (3.34 to 3.39) | 1.00 (Reference) |
| Pre-vaccination | 21,522 | 1.96 (1.94 to 1.99) | 0.80 (0.79 to 0.81) | 2,040 | 2.36 (2.26 to 2.47) | 0.79 (0.76 to 0.83) |
| Post-vaccination | 23,490 | 1.77 (1.74 to 1.79) | 0.75 (0.74 to 0.76) | 1,599 | 1.83 (1.74 to 1.92) | 0.88 (0.84 to 0.92) |
| **Perceptual disturbances and psychoses** | |  |  |  |  |  |
| Baseline | 51,189 | 3.82 (3.78 to 3.85) | 1.00 (Reference) | 13,629 | 3.29 (3.23 to 3.34) | 1.00 (Reference) |
| Pre-vaccination | 2,444 | 1.31 (1.26 to 1.36) | 0.72 (0.69 to 0.75) | 825 | 4.01 (3.75 to 4.29) | 1.38 (1.29 to 1.47) |
| Post-vaccination | 2,796 | 1.30 (1.25 to 1.34) | 0.72 (0.69 to 0.75) | 325 | 1.51 (1.36 to 1.69) | 0.72 (0.65 to 0.80) |
| **Aggression and behavioral disturbances** | |  |  |  |  |  |
| Baseline | 23,711 | 3.62 (3.57 to 3.67) | 1.00 (Reference) | 4,215 | 3.24 (3.15 to 3.34) | 1.00 (Reference) |
| Pre-vaccination | 1,430 | 1.84 (1.74 to 1.93) | 0.93 (0.88 to 0.98) | 61 | 3.11 (2.42 to 4.00) | 1.01 (0.80 to 1.28) |
| Post-vaccination | 1,832 | 1.82 (1.74 to 1.91) | 0.94 (0.89 to 0.99) | 60 | 2.98 (2.32 to 3.84) | 1.27 (0.99 to 1.61) |
| **Cognitive impairments** |  |  |  |  |  |  |
| Baseline | 162,017 | 3.41 (3.40 to 3.43) | 1.00 (Reference) | 56,321 | 3.37 (3.35 to 3.40) | 1.00 (Reference) |
| Pre-vaccination | 22,403 | 3.18 (3.14 to 3.22) | 0.78 (0.77 to 0.79) | 2,564 | 2.51 (2.42 to 2.61) | 0.76 (0.73 to 0.78) |
| Post-vaccination | 23,921 | 2.42 (2.39 to 2.45) | 0.66 (0.65 to 0.67) | 1,756 | 1.71 (1.63 to 1.79) | 0.72 (0.69 to 0.75) |
| **Sleep disorder** |  |  |  |  |  |  |
| Baseline | 219,510 | 3.61 (3.59 to 3.62) | 1.00 (Reference) | 44,300 | 3.36 (3.32 to 3.39) | 1.00 (Reference) |
| Pre-vaccination | 21,361 | 2.17 (2.14 to 2.20) | 0.82 (0.80 to 0.83) | 2,141 | 2.31 (2.21 to 2.41) | 0.78 (0.75 to 0.81) |
| Post-vaccination | 25,943 | 2.21 (2.18 to 2.23) | 0.86 (0.85 to 0.87) | 2,323 | 2.48 (2.38 to 2.59) | 1.18 (1.13 to 1.23) |

**Abbreviations**: CI, confidence interval.

Pre-vaccination and post-vaccination were defined as 21 days before and after the vaccination date, respectively.

^*^mRNA vaccinations included BNT162b2 and mRNA-1273, and viral vector vaccinations included ChAdOx1 nCoV-19 and Ad.26. COV2.S, respectively.

^†^ incidence rate was calculated per 1,000 person-days.

^‡^ Season and infection of COVID-19 were adjusted with a time-varying approach.

**Supplementary table 7.** Risk of psychiatric adverse events following COVID-19 vaccination in South Korea stratified by individual vaccines

|  | **No. of**  **events** | **Incidence rate^*^**  **(95% CI)** | **Incidence rate ratio^†^**  **(95% CI)** |
| --- | --- | --- | --- |
| **BNT162b2** | |  |  |
| **Anxiety and nervousness** | |  |  |
| Baseline | 244,894 | 3.52 (3.51 to 3.54) | 1.00 (Reference) |
| Pre-vaccination | 24,766 | 2.38 (2.35 to 2.41) | 0.87 (0.86 to 0.88) |
| Post-vaccination | 30,457 | 2.38 (2.36 to 2.41) | 0.91 (0.90 to 0.92) |
| **Mood disorders** |  |  |  |
| Baseline | 224,128 | 3.67 (3.65 to 3.68) | 1.00 (Reference) |
| Pre-vaccination | 18,309 | 2.05 (2.02 to 2.08) | 0.92 (0.90 to 0.94) |
| Post-vaccination | 19,930 | 1.80 (1.78 to 1.83) | 0.92 (0.90 to 0.94) |
| **Perceptual disturbances and psychoses** | |  |  |
| Baseline | 41,323 | 3.76 (3.72 to 3.80) | 1.00 (Reference) |
| Pre-vaccination | 2,116 | 1.45 (1.39 to 1.51) | 1.44 (1.39 to 1.50) |
| Post-vaccination | 2,381 | 1.39 (1.34 to 1.45) | 0.86 (0.81 to 0.90) |
| **Aggression and behavioral disturbances** | |  |  |
| Baseline | 20,874 | 3.61 (3.57 to 3.66) | 1.00 (Reference) |
| Pre-vaccination | 1,230 | 1.86 (1.76 to 1.97) | 0.94 (0.89 to 1.00) |
| Post-vaccination | 1,526 | 1.76 (1.68 to 1.85) | 0.91 (0.86 to 0.96) |
| **Cognitive impairments** |  |  |  |
| Baseline | 156,211 | 3.40 (3.39 to 3.42) | 1.00 (Reference) |
| Pre-vaccination | 21,841 | 3.24 (3.20 to 3.28) | 1.09 (0.98 to 1.21) |
| Post-vaccination | 23,276 | 2.43 (2.40 to 2.46) | 0.95 (0.85 to 1.06) |
| **Sleep disorders** |  |  |  |
| Baseline | 181,034 | 3.59 (3.57 to 3.60) | 1.00 (Reference) |
| Pre-vaccination | 17,987 | 2.25 (2.22 to 2.29) | 0.91 (0.89 to 0.93) |
| Post-vaccination | 21,753 | 2.23 (2.20 to 2.26) | 1.03 (1.01 to 1.05) |
| **mRNA-1273** | |  |  |
| **Anxiety and nervousness** | |  |  |
| Baseline | 88,977 | 3.45 (3.43 to 3.47) | 1.00 (Reference) |
| Pre-vaccination | 5,181 | 2.04 (1.99 to 2.10) | 1.00 (0.97 to 1.03) |
| Post-vaccination | 6,468 | 2.34 (2.28 to 2.39) | 1.18 (1.15 to 1.21) |
| **Mood disorders** |  |  |  |
| Baseline | 79,626 | 3.55 (3.53 to 3.58) | 1.00 (Reference) |
| Pre-vaccination | 3,213 | 1.59 (1.53 to 1.64) | 0.94 (0.91 to 0.98) |
| Post-vaccination | 3,560 | 1.58 (1.53 to 1.63) | 0.97 (0.94 to 1.01) |
| **Perceptual disturbances and psychoses** | |  |  |
| Baseline | 19,159 | 3.62 (3.57 to 3.67) | 1.00 (Reference) |
| Pre-vaccination | 328 | 0.81 (0.73 to 0.90) | 0.81 (0.72 to 0.90) |
| Post-vaccination | 415 | 0.92 (0.84 to 1.01) | 0.96 (0.86 to 1.06) |
| **Aggression and behavioral disturbances** | |  |  |
| Baseline | 6,617 | 3.40 (3.32 to 3.49) | 1.00 (Reference) |
| Pre-vaccination | 200 | 1.70 (1.48 to 1.95) | 0.95 (0.82 to 1.09) |
| Post-vaccination | 306 | 2.19 (1.96 to 2.45) | 1.25 (1.11 to 1.41) |
| **Cognitive impairments** |  |  |  |
| Baseline | 40,177 | 3.30 (3.26 to 3.33) | 1.00 (Reference) |
| Pre-vaccination | 562 | 1.90 (1.75 to 2.07) | 1.46 (1.35 to 1.59) |
| Post-vaccination | 645 | 2.11 (1.95 to 2.28) | 1.73 (1.60 to 1.87) |
| **Sleep disorders** |  |  |  |
| Baseline | 63,744 | 3.50 (3.47 to 3.53) | 1.00 (Reference) |
| Pre-vaccination | 3,374 | 1.83 (1.77 to 1.89) | 0.96 (0.93 to 1.00) |
| Post-vaccination | 4,190 | 2.11 (2.05 to 2.18) | 1.15 (1.11 to 1.18) |
| **ChAdOx1 nCoV-19** | |  |  |
| **Anxiety and nervousness** | |  |  |
| Baseline | 55,568 | 3.31 (3.29 to 3.34) | 1.00 (Reference) |
| Pre-vaccination | 2,556 | 2.51 (2.41 to 2.61) | 0.82 (0.79 to 0.86) |
| Post-vaccination | 2,756 | 2.68 (2.58 to 2.78) | 1.19 (1.14 to 1.23) |
| **Mood disorders** |  |  |  |
| Baseline | 50,717 | 3.36 (3.33 to 3.39) | 1.00 (Reference) |
| Pre-vaccination | 1,971 | 2.37 (2.27 to 2.48) | 0.79 (0.76 to 0.83) |
| Post-vaccination | 1,533 | 1.82 (1.73 to 1.91) | 0.87 (0.83 to 0.92) |
| **Perceptual disturbances and psychoses** | |  |  |
| Baseline | 13,389 | 3.28 (3.23 to 3.34) | 1.00 (Reference) |
| Pre-vaccination | 815 | 4.09 (3.82 to 4.38) | 1.39 (1.30 to 1.48) |
| Post-vaccination | 306 | 1.47 (1.31 to 1.64) | 0.69 (0.62 to 0.77) |
| **Aggression and behavioral disturbances** | |  |  |
| Baseline | 4,120 | 3.24 (3.14 to 3.34) | 1.00 (Reference) |
| Pre-vaccination | 56 | 3.30 (2.54 to 4.29) | 1.07 (0.84 to 1.37) |
| Post-vaccination | 48 | 2.74 (2.06 to 3.63) | 1.13 (0.86 to 1.48) |
| **Cognitive impairments** |  |  |  |
| Baseline | 56,010 | 3.37 (3.35 to 3.40) | 1.00 (Reference) |
| Pre-vaccination | 2,554 | 2.53 (2.43 to 2.63) | 0.76 (0.73 to 0.78) |
| Post-vaccination | 1,734 | 1.70 (1.62 to 1.78) | 0.71 (0.68 to 0.75) |
| **Sleep disorders** |  |  |  |
| Baseline | 43,247 | 3.36 (3.32 to 3.90) | 1.00 (Reference) |
| Pre-vaccination | 2,074 | 2.31 (2.21 to 2.41) | 0.78 (0.74 to 0.81) |
| Post-vaccination | 2,231 | 2.46 (2.36 to 2.56) | 1.17 (1.12 to 1.22) |
| **Ad26.COV2-S** | |  |  |
| **Anxiety and nervousness** | |  |  |
| Baseline | 37,073 | 3.24 (3.20 to 3.27) | 1.00 (Reference) |
| Pre-vaccination | 92 | 2.37 (1.94 to 2.91) | 0.81 (0.66 to 0.99) |
| Post-vaccination | 148 | 3.84 (3.27 to 4.51) | 1.90 (1.63 to 2.22) |
| **Mood disorders** |  |  |  |
| Baseline | 34,597 | 3.24 (3.21 to 3.28) | 1.00 (Reference) |
| Pre-vaccination | 69 | 2.21 (1.75 to 2.80) | 0.83 (0.66 to 1.05) |
| Post-vaccination | 66 | 2.12 (1.67 to 2.70) | 1.27 (1.01 to 1.61) |
| **Perceptual disturbances and psychoses** | |  |  |
| Baseline | 9,533 | 3.24 (3.18 to 3.31) | 1.00 (Reference) |
| Pre-vaccination | 10 | 1.55 (0.83 to 2.87) | 0.88 (0.48 to 1.62) |
| Post-vaccination | 19 | 2.95 (1.88 to 4.62) | 2.98 (1.93 to 4.61) |
| **Aggression and behavioral disturbances** | |  |  |
| Baseline | 3,875 | 3.24 (3.14 to 3.34) | 1.00 (Reference) |
| Pre-vaccination | 5 | 1.89 (0.79 to 4.54) | 0.61 (0.26 to 1.44) |
| Post-vaccination | 12 | 4.64 (2.63 to 8.16) | 2.52 (1.48 to 4.28) |
| **Cognitive impairments** |  |  |  |
| Baseline | 34,682 | 3.24 (3.20 to 3.27) | 1.00 (Reference) |
| Pre-vaccination | 10 | 1.22 (0.66 to 2.26) | 0.67 (0.37 to 1.24) |
| Post-vaccination | 22 | 2.69 (1.77 to 4.08) | 2.39 (1.60 to 3.57) |
| **Sleep disorders** |  |  |  |
| Baseline | 26,321 | 3.24 (3.20 to 3.28) | 1.00 (Reference) |
| Pre-vaccination | 67 | 2.34 (1.84 to 2.98) | 0.88 (0.70 to 1.11) |
| Post-vaccination | 92 | 3.23 (2.64 to 3.97) | 1.80 (1.48 to 2.19) |

**Abbreviations**: CI, confidence interval.

Pre-vaccination and post-vaccination were defined as 21 days before and after the vaccination date, respectively.

^*^Incidence rate was calculated per 1,000 person-days.

^†^Season and infection of COVID-19 were adjusted with a time-varying approach.

**Supplementary table 8.** Risk of psychiatric adverse events following COVID-19 vaccination in South Korea, stratified by vaccination dose

|  | **No. of**  **events** | **Incidence rate^*^**  **(95% CI)** | **Incidence rate ratio^†^**  **(95% CI)** |
| --- | --- | --- | --- |
| **Anxiety and nervousness** | |  |  |
| Baseline | 429,906 | 3.58 (3.57 to 3.59) | 1.00 (Reference) |
| Pre-vaccination | 54,449 | 2.35 (2.33 to 2.37) | 0.82 (0.82 to 0.83) |
| Post-vaccination (1^st^ dose) | 34,134 | 2.75 (2.72 to 2.78) | 0.96 (0.95 to 0.97) |
| Post-vaccination (2^nd^ dose) | 20,929 | 2.71 (2.68 to 2.74) | 0.94 (0.93 to 0.95) |
| Post-vaccination (3^rd^ dose) | 5,365 | 2.73 (2.69 to 2.77) | 0.95 (0.91 to 0.99) |
| **Mood disorders** |  |  |  |
| Baseline | 381,345 | 3.75 (3.74 to 3.76) | 1.00 (Reference) |
| Pre-vaccination | 39,767 | 2.09 (2.07 to 2.11) | 0.76 (0.75 to 0.76) |
| Post-vaccination (1^st^ dose) | 20,699 | 2.09 (2.06 to 2.12) | 0.76 (0.75 to 0.77) |
| Post-vaccination (2^nd^ dose) | 13,194 | 1.72 (1.69 to 1.75) | 0.74 (0.73 to 0.75) |
| Post-vaccination (3^rd^ dose) | 3,314 | 1.61 (1.58 to 1.64) | 0.71 (0.68 to 0.74) |
| **Perceptual disturbances and psychoses** | |  |  |
| Baseline | 72,161 | 3.59 (3.56 to 3.61) | 1.00 (Reference) |
| Pre-vaccination | 11,564 | 3.15 (3.10 to 3.21) | 1.25 (1.23 to 1.28) |
| Post-vaccination (1^st^ dose) | 3,978 | 1.72 (1.67 to 1.77) | 0.74 (0.72 to 0.76) |
| Post-vaccination (2^nd^ dose) | 1,640 | 1.61 (1.56 to 1.66) | 0.71 (0.68 to 0.74) |
| Post-vaccination (3^rd^ dose) | 539 | 1.49 (1.40 to 1.58) | 0.66 (0.59 to 0.73) |
| **Aggression and behavioral disturbances** | |  |  |
| Baseline | 26,703 | 3.61 (3.56 to 3.65) | 1.00 (Reference) |
| Pre-vaccination | 2,026 | 2.04 (1.96 to 2.14) | 0.90 (0.86 to 0.94) |
| Post-vaccination (1^st^ dose) | 1,247 | 2.01 (1.90 to 2.12) | 0.95 (0.90 to 1.00) |
| Post-vaccination (2^nd^ dose) | 958 | 1.87 (1.75 to 1.99) | 0.92 (0.86 to 0.98) |
| Post-vaccination (3^rd^ dose) | 141 | 1.79 (1.51 to 2.07) | 0.90 (0.78 to 1.02) |
| **Cognitive impairments** |  |  |  |
| Baseline | 267,006 | 3.56 (3.55 to 3.57) | 1.00 (Reference) |
| Pre-vaccination | 42,935 | 2.84 (2.81 to 2.87) | 0.74 (0.74 to 0.75) |
| Post-vaccination (1^st^ dose) | 21,506 | 2.19 (2.16 to 2.22) | 0.68 (0.67 to 0.69) |
| Post-vaccination (2^nd^ dose) | 12,860 | 2.08 (2.04 to 2.12) | 0.65 (0.64 to 0.66) |
| Post-vaccination (3^rd^ dose) | 4,126 | 2.89 (2.86 to 2.92) | 0.75 (0.73 to 0.77) |
| **Sleep disorders** |  |  |  |
| Baseline | 343,440 | 3.66 (3.64 to 3.67) | 1.00 (Reference) |
| Pre-vaccination | 43,385 | 2.23 (2.21 to 2.26) | 0.76 (0.76 to 0.77) |
| Post-vaccination (1^st^ dose) | 25,427 | 2.76 (2.73 to 2.79) | 0.95 (0.94 to 0.96) |
| Post-vaccination (2^nd^ dose) | 15,933 | 2.14 (2.11 to 2.17) | 0.87 (0.86 to 0.88) |
| Post-vaccination (3^rd^ dose) | 5,821 | 2.01 (1.97 to 2.05) | 0.80 (0.78 to 0.82) |

**Abbreviations**: CI, confidence interval.

Pre-vaccination and post-vaccination were defined as 21 days before and after the vaccination date, respectively.

^*^Incidence rate was calculated per 1,000 person-days.

^†^Season and infection of COVID-19 were adjusted with a time-varying approach.

**Supplementary table 9.** Sensitivity analyses for the risk of psychiatric adverse events following COVID-19 vaccination in South Korea: Excluding death cases during the study period

|  | **No. of**  **events** | **Incidence rate^*^**  **(95% CI)** | **Incidence rate ratio^†^**  **(95% CI)** |
| --- | --- | --- | --- |
| **Anxiety and nervousness** | |  |  |
| Baseline | 426,703 | 3.58 (3.57 to 3.59) | 1.00 (Reference) |
| Pre-vaccination | 54,200 | 2.34 (2.32 to 2.36) | 0.82 (0.81 to 0.83) |
| Post-vaccination | 60,171 | 2.41 (2.39 to 2.43) | 0.95 (0.94 to 0.96) |
| **Mood disorders** |  |  |  |
| Baseline | 376,486 | 3.76 (3.75 to 3.77) | 1.00 (Reference) |
| Pre-vaccination | 39,315 | 2.08 (2.06 to 2.10) | 0.75 (0.74 to 0.76) |
| Post-vaccination | 36,874 | 1.77 (1.76 to 1.79) | 0.75 (0.74 to 0.76) |
| **Perceptual disturbances and psychoses** | |  |  |
| Baseline | 70,957 | 3.60 (3.57 to 3.62) | 1.00 (Reference) |
| Pre-vaccination | 11,411 | 3.13 (3.08 to 3.19) | 1.25 (1.23 to 1.28) |
| Post-vaccination | 6,085 | 1.54 (1.50 to 1.57) | 0.72 (0.71 to 0.74) |
| **Aggression and behavioral disturbances** | |  |  |
| Baseline | 26,548 | 3.61 (3.57 to 3.65) | 1.00 (Reference) |
| Pre-vaccination | 2,020 | 2.04 (1.96 to 2.13) | 0.90 (0.86 to 0.94) |
| Post-vaccination | 2,333 | 1.93 (1.86 to 2.01) | 0.93 (0.89 to 0.97) |
| **Cognitive impairments** |  |  |  |
| Baseline | 249,177 | 3.59 (3.57 to 3.60) | 1.00 (Reference) |
| Pre-vaccination | 41,576 | 2.81 (2.78 to 2.84) | 0.74 (0.73 to 0.75) |
| Post-vaccination | 37,548 | 2.19 (2.16 to 2.21) | 0.68 (0.67 to 0.69) |
| **Sleep disorders** |  |  |  |
| Baseline | 339,208 | 3.66 (3.65 to 3.67) | 1.00 (Reference) |
| Pre-vaccination | 43,115 | 2.23 (2.21 to 2.25) | 0.76 (0.75 to 0.77) |
| Post-vaccination | 46,912 | 2.27 (2.25 to 2.29) | 0.90 (0.89 to 0.91) |

**Abbreviations**: CI, confidence interval.

Pre-vaccination and post-vaccination were defined as 21 days before and after the vaccination date, respectively.

^*^Incidence rate was calculated per 1,000 person-days.

^†^Season and infection of COVID-19 were adjusted with a time-varying approach.

**Supplementary table 10.** Sensitivity analyses for the risk of psychiatric adverse events following COVID-19 vaccination in South Korea: Excluding COVID-19 cases during the study period

|  | **No. of**  **events** | **Incidence rate^*^**  **(95% CI)** | **Incidence rate ratio^†^**  **(95% CI)** |
| --- | --- | --- | --- |
| **Anxiety and nervousness** | |  |  |
| Baseline | 425,530 | 3.58 (3.57 to 3.59) | 1.00 (Reference) |
| Pre-vaccination | 54,008 | 2.35 (2.33 to 2.37) | 0.82 (0.82 to 0.83) |
| Post-vaccination | 59,897 | 2.41 (2.39 to 2.43) | 0.95 (0.95 to 0.96) |
| **Mood disorders** |  |  |  |
| Baseline | 377,358 | 3.75 (3.74 to 3.76) | 1.00 (Reference) |
| Pre-vaccination | 39,392 | 2.09 (2.07 to 2.11) | 0.76 (0.75 to 0.76) |
| Post-vaccination | 36,839 | 1.78 (1.76 to 1.80) | 0.75 (0.75 to 0.76) |
| **Perceptual disturbances and psychoses** | |  |  |
| Baseline | 71,415 | 3.59 (3.57 to 3.62) | 1.00 (Reference) |
| Pre-vaccination | 11,389 | 3.13 (3.08 to 3.19) | 1.25 (1.23 to 1.27) |
| Post-vaccination | 6,096 | 1.54 (1.50 to 1.58) | 0.72 (0.71 to 0.74) |
| **Aggression and behavioral disturbances** | |  |  |
| Baseline | 26,353 | 3.61 (3.57 to 3.65) | 1.00 (Reference) |
| Pre-vaccination | 2,006 | 2.04 (1.96 to 2.14) | 0.90 (0.86 to 0.94) |
| Post-vaccination | 2,323 | 1.94 (1.86 to 2.02) | 0.93 (0.89 to 0.98) |
| **Cognitive impairments** |  |  |  |
| Baseline | 264,123 | 3.56 (3.55 to 3.58) | 1.00 (Reference) |
| Pre-vaccination | 42,538 | 2.84 (2.81 to 2.87) | 0.74 (0.74 to 0.75) |
| Post-vaccination | 38,158 | 2.19 (2.17 to 2.21) | 0.68 (0.67 to 0.69) |
| **Sleep disorders** |  |  |  |
| Baseline | 339,651 | 3.66 (3.65 to 3.67) | 1.00 (Reference) |
| Pre-vaccination | 42,934 | 2.23 (2.21 to 2.25) | 0.76 (0.75 to 0.77) |
| Post-vaccination | 46,678 | 2.27 (2.25 to 2.29) | 0.90 (0.89 to 0.91) |

**Abbreviations**: CI, confidence interval.

Pre-vaccination and post-vaccination were defined as 21 days before and after the vaccination date, respectively.

^*^Incidence rate was calculated per 1,000 person-days.

^†^Season and infection of COVID-19 were adjusted with a time-varying approach.

**Supplementary table 11.** Sensitivity analyses for the risk of psychiatric adverse events following COVID-19 vaccination in South Korea: Splitting the risk interval

|  | **No. of**  **events** | **Incidence rate^*^**  **(95% CI)** | **Incidence rate ratio^†^**  **(95% CI)** |
| --- | --- | --- | --- |
| **Anxiety and nervousness** | |  |  |
| Baseline | 429,906 | 3.58 (3.57 to 3.59) | 1.00 (Reference) |
| Pre-vaccination | 54,449 | 2.35 (2.33 to 2.37) | 0.82 (0.82 to 0.83) |
| Post-vaccination (1-7 days after) | 17,922 | 2.32 (2.28 to 2.35) | 0.95 (0.94 to 0.97) |
| Post-vaccination (8-14 days after) | 21,547 | 2.32 (2.29 to 2.35) | 0.88 (0.87 to 0.89) |
| Post-vaccination (15-21 days after) | 20,959 | 2.61 (2.57 to 2.64) | 1.03 (1.02 to 1.05) |
| **Mood disorders** |  |  |  |
| Baseline | 381,345 | 3.75 (3.74 to 3.76) | 1.00 (Reference) |
| Pre-vaccination | 39,767 | 2.09 (2.07 to 2.11) | 0.76 (0.75 to 0.76) |
| Post-vaccination (1-7 days after) | 11,830 | 1.78 (1.75 to 1.81) | 0.76 (0.75 to 0.77) |
| Post-vaccination (8-14 days after) | 11,209 | 1.75 (1.72 to 1.78) | 0.79 (0.77 to 0.80) |
| Post-vaccination (15-21 days after) | 14,168 | 1.81 (1.78 to 1.84) | 0.73 (0.72 to 0.74) |
| **Perceptual disturbances and psychoses** | |  |  |
| Baseline | 72,161 | 3.59 (3.56 to 3.61) | 1.00 (Reference) |
| Pre-vaccination | 11,564 | 3.15 (3.10 to 3.21) | 1.25 (1.23 to 1.28) |
| Post-vaccination (1-7 days after) | 2,295 | 1.75 (1.68 to 1.83) | 0.78 (0.75 to 0.81) |
| Post-vaccination (8-14 days after) | 1,838 | 1.45 (1.39 to 1.52) | 0.72 (0.68 to 0.75) |
| Post-vaccination (15-21 days after) | 2,024 | 1.43 (1.37 to 1.49) | 0.67 (0.64 to 0.70) |
| **Aggression and behavioral disturbances** | |  |  |
| Baseline | 26,703 | 3.61 (3.56 to 3.65) | 1.00 (Reference) |
| Pre-vaccination | 2,026 | 2.04 (1.96 to 2.14) | 0.90 (0.86 to 0.94) |
| Post-vaccination (1-7 days after) | 937 | 1.95 (1.81 to 2.10) | 0.94 (0.87 to 1.01) |
| Post-vaccination (8-14 days after) | 696 | 1.97 (1.82 to 2.10) | 0.96 (0.89 to 1.04) |
| Post-vaccination (15-21 days after) | 713 | 1.91 (1.79 to 2.04) | 0.91 (0.85 to 0.97) |
| **Cognitive impairments** |  |  |  |
| Baseline | 267,006 | 3.56 (3.55 to 3.57) | 1.00 (Reference) |
| Pre-vaccination | 42,935 | 2.84 (2.81 to 2.87) | 0.74 (0.74 to 0.75) |
| Post-vaccination (1-7 days after) | 12,607 | 2.47 (2.43 to 2.51) | 0.79 (0.77 to 0.80) |
| Post-vaccination (8-14 days after) | 9,943 | 2.01 (1.97 to 2.05) | 0.69 (0.68 to 0.71) |
| Post-vaccination (15-21 days after) | 15,942 | 2.12 (2.09 to 2.16) | 0.60 (0.59 to 0.61) |
| **Sleep disorders** |  |  |  |
| Baseline | 343,440 | 3.66 (3.64 to 3.67) | 1.00 (Reference) |
| Pre-vaccination | 43,385 | 2.23 (2.21 to 2.26) | 0.76 (0.76 to 0.77) |
| Post-vaccination (1-7 days after) | 17,846 | 2.67 (2.63 to 2.71) | 1.06 (1.05 to 1.08) |
| Post-vaccination (8-14 days after) | 13,280 | 2.07 (2.03 to 2.10) | 0.86 (0.85 to 0.88) |
| Post-vaccination (15-21 days after) | 16,055 | 2.10 (2.07 to 2.13) | 0.80 (0.78 to 0.81) |

**Abbreviations**: CI, confidence interval.

Pre-vaccination and post-vaccination were defined as 21 days before and after the date of vaccination, respectively, and the post-vaccination period was divided into 3 intervals of 7 days.

^*^Incidence rate was calculated per 1,000 person-days.

^†^Season and infection of COVID-19 were adjusted with a time-varying approach.

**Supplementary table 12.** The number of patients diagnosed with diagnostic code in the total study cohort of our study

|  | **F06** | **F19** | **F20** | **F91** | **F93** |
| --- | --- | --- | --- | --- | --- |
| No. of patients | 169,565  (1.44%) | 1,365  (0.01%) | 43,527  (0.37%) | 2,025  (0.02%) | 1,117  (0.01%) |

The proportion was calculated using the total number of study participants as the denominator.

F06: Other mental disorders due to brain damage and dysfunction and to physical disease

F19: Mental and behavioural disorders due to multiple drug use and use of other psychoactive substances

F20: Schizophrenia

F91: Conduct disorders

**Supplementary table 13.** The number of patients according to 4-digit ICD-10 codes among those diagnosed with F06

| **Diagnostic code** | | **No. of patients** |
| --- | --- | --- |
| F06.0 | Organic hallucinosis | 202 (0.1) |
| F06.1 | Organic catatonic disorder | 423 (0.2) |
| F06.2 | Organic delusional [schizophrenia-like] disorder | 1,228 (0.7) |
| F06.3 | Organic mood [affective] disorders | 1,202 (0.7) |
| F06.4 | Organic anxiety disorder | 2,774 (1.6) |
| F06.5 | Organic dissociative disorder | 25 (0.0) |
| F06.6 | Organic emotionally labile [asthenic] disorder | 858 (0.5) |
| F06.7 | Mild cognitive disorder | 156,202 (92.1) |
| F06.8 | Other specified mental disorders due to brain damage and dysfunction and to physical disease | 2,056 (1.2) |
| F06.9 | Unspecified mental disorder due to brain damage and dysfunction and to physical disease | 7,246 (4.3) |

The proportion was calculated using the total number of patients diagnosed with F06 (Other mental disorders due to brain damage and dysfunction and to physical disease; no. of patients: 169,565) as the denominator.
